# How can clinicians choose between conflicting and discordant systematic reviews? A replication study of the Jadad algorithm

**DOI:** 10.1186/s12874-022-01750-2

**Published:** 2022-10-26

**Authors:** C Lunny, Sai Surabi Thirugnanasampanthar, S Kanji, N Ferri, D Pieper, S Whitelaw, S Tasnim, H Nelson, EK Reid, Jia He (Janet) Zhang, Banveer Kalkat, Yuan Chi, Reema Abdoulrezzak, Di Wen Zheng, Lindy R.S. Pangka, Dian (Xin Ran) Wang, Parisa Safavi, Anmol Sooch, Kevin T. Kang, Andrea C, Tricco

**Affiliations:** 1 Unity Health Toronto and the Cochrane Hypertension Review Group, St Michael’s Hospital, University of British Columbia, V6T 1Z3 Vancouver, BC Canada; 2 Epidemiology Division, Institute for Health Policy, Management, and Evaluation, Dalla Lana School of Public Health, University of Toronto, Toronto, ON Canada; 3 The Ottawa Hospital, Ottawa Hospital Research Institute, Ottawa, Canada; 4 Division of Occupational Medicine, IRCCS Azienda Ospedaliero-Universitaria di Bologna, Bologna, Italy; 5 Department of Biomedical and Neuromotor Sciences (DIBINEM), Alma Mater Studiorum, University of Bologna, 40138 Bologna, Italy; 6 Faculty of Health Sciences Brandenburg, Brandenburg Medical School Theodor Fontane, Institute for Health Services and Health System Research, Rüdersdorf, Germany; 7 Center for Health Services Research, Brandenburg Medical School Theodor Fontane, Rüdersdorf, Germany; 8 Faculty of Medicine and Health Sciences, McGill University, Montreal, QC Canada; 9 Cochrane Hypertension Review Group, University of British Columbia, 2176 Health Science Mall, Vancouver, BC V6T 1Z3 Canada; 10 Faculty of Health Sciences, Queen’s University, Kingston, ON Canada; 11 Nova Scotia Health, Halifax, NS Canada; 12 Faculty of Science, University of British Columbia, Vancouver, BC Canada; 13 Faculty of Pharmaceutical Sciences, University of British Columbia, Vancouver, BC Canada; 14 Beijing Yealth Technology Co., Ltd, Beijing, China; 15 Cochrane Campbell Global Ageing Partnership, London, United Kingdom; 16 Knowledge Translation Program, Li Ka Shing Knowledge Institute, St. Michael’s Hospital, Unity Health Toronto, 209 Victoria St, M5B 1T8 Toronto, ON Canada; 17 Epidemiology Division, Institute for Health Policy, Management, and Evaluation, Dalla Lana School of Public Health, University of Toronto, 155 College St Room 500, M5T 3M7 Toronto, ON Canada; 18 Queen’s Collaboration for Health Care Quality Joanna Briggs Institute Centre of Excellence, School of Nursing, Queen’s University, 99 University Ave, K7L 3N6 Kingston, ON Canada

**Keywords:** Discordance, Overviews of reviews, Overlapping, Systematic reviews, Meta-analyses, Conflicting, Discordant, Agreement, Concordant, Replication, Knowledge synthesis, Evidence synthesis

## Abstract

**Introduction:**

The exponential growth of published systematic reviews (SRs) presents challenges for decision makers seeking to answer clinical, public health or policy questions. In 1997, an algorithm was created by Jadad et al. to choose the best SR across multiple. Our study aims to replicate author assessments using the Jadad algorithm to determine: (i) if we chose the same SR as the authors; and (ii) if we reach the same results.

**Methods:**

We searched MEDLINE, Epistemonikos, and Cochrane Database of SRs. We included any study using the Jadad algorithm. We used consensus building strategies to operationalise the algorithm and to ensure a consistent approach to interpretation.

**Results:**

We identified 21 studies that used the Jadad algorithm to choose one or more SRs. In 62% (13/21) of cases, we were unable to replicate the Jadad assessment and ultimately chose a different SR than the authors. Overall, 18 out of the 21 (86%) independent Jadad assessments agreed in direction of the findings despite 13 having chosen a different SR.

**Conclusions:**

Our results suggest that the Jadad algorithm is not reproducible between users as there are no prescriptive instructions about how to operationalise the algorithm. In the absence of a validated algorithm, we recommend that healthcare providers, policy makers, patients and researchers address conflicts between review findings by choosing the SR(s) with meta-analysis of RCTs that most closely resemble their clinical, public health, or policy question, are the most recent, comprehensive (i.e. number of included RCTs), and at the lowest risk of bias.

**Supplementary Information:**

The online version contains supplementary material available at 10.1186/s12874-022-01750-2.

## Highlights

This is the first empirical study to replicate Jadad algorithm assessments to evaluate discordance across systematic reviews.

In 62% (13/21) of cases, we were unable to replicate the Jadad algorithm assessment and ultimately chose a different systematic review than the authors.

When assessing systematic reviews using the Jadad algorithm, some steps of the Jadad algorithm were vague in description, making it difficult to operationalise, interpret, and use.

The Jadad algorithm has several limitations as it does not account for the last literature search of the systematic review and publication recency of included trials.

To assess discordance in the absence of an algorithm, we recommend decision makers consider relevance (objectives that most closely resemble their clinical question), recency (dates of search), comprehensiveness (most trials), and risk of bias (lowest risk of bias SR) when choosing one systematic review across multiple.

**Supplementary Information:**

The online version contains supplementary material available at 10.1186/s12874-022-01750-2.

## 1.0 Background

Keeping up with current research for a practicing clinician or policy maker is a monumental task. Global research output is increasing exponentially [1] as is the quantity of published systematic reviews being produced yearly [2–4]. Systematic reviews (SRs) help clinicians navigate complex clinical topics by summarising large numbers of primary studies. Between 2000 and 2019, the number of SRs increased more than 20-fold, with 80 SRs published per day [5].

The increase in the number of SRs means that overlapping and redundant reviews are increasingly found on the same clinical, public health, or policy question of interest. Bolland et al. found 24 SRs on vitamin D supplements for prevention of bone fractures, and many of these contained conflicting results based on diverse methodological choices and differing included primary studies [6]. When encountering multiple SRs on the same question, clinicians and policy makers may be confused and unable to formulate a conclusive answer to their question [7].

To surmount this challenge, an algorithm was published in 1997 by Jadad et al. [8] to aid healthcare providers and policy makers select the “best evidence” SR(s) across multiple reviews of randomized controlled trials (RCTs) with meta-analysis addressing the same or a very similar therapeutic question, with results that “diverge” or conflict. If the reviews do not address the same question, then no further assessment is needed, and the decision maker simply chooses the review “closest to the problem to be solved”. If, however, two or more similar reviews are identified that are discordant, then a hierarchy of steps is followed to choose the best evidence.

Overviews of reviews (otherwise termed umbrella reviews, meta-reviews, etc.) were developed to summarise the results of SRs and can help make sense of potentially conflicting or discordant results [9–12]. However, a new type of study emerged, with more focused objectives than overviews of reviews, aiming to assess discordance in results across multiple similar SRs. The more focused discordance studies are often called ‘reviews of overlapping meta-analyses’, ‘reviews of discordant SRs’, or ‘reviews of discordant meta-analyses’. In this paper, we will call them “Discordant Reviews” for clarity and to distinguish them from other types of reviews and ‘overviews of reviews’. In our study, we define discordance as when SRs with identical or nearly identical clinical, public health, or policy eligibility criteria (as expressed in PICO [population, intervention, comparison, outcome] elements) report different results for the same outcome. We define discordant results, and authors’ interpretation of the results of SRs, as differences in results of SRs based on the methodological decisions authors make, or different interpretations or judgments about the results [11].

An empirical and systematic mapping study identified formal and informal approaches for dealing with multiple overlapping SRs with discordant results [10, 11]. One approach was to specify methodological criteria to select a single, most representative SR (e.g., select the highest quality and most comprehensive) [11]. Other identified approaches were to examine and record discordance and use tools (i.e. Jadad algorithm [8]) or decision rules to aid in the selection of one SR [10, 11]. This systematic mapping study identified only one tool to assess discordance, namely the Jadad algorithm, and given that there are no other options available to assess discordance across SRs, it will continue to be used by researchers. Indeed it is still being commonly used today [13–15].

The Jadad algorithm has not been universally adopted and has been inconsistently applied [16–18]. We believe our research is unique as we did not identify any study aiming to replicate the Jadad algorithm. Our study objectives were to: identify Discordant Reviews that used the Jadad algorithm to address discordance amongst SRs with meta-analysis of RCTs; replicate Jadad assessments done by authors to determine if the same SR(s) would be chosen, and explore reasons for reproducibility or lack thereof.

## 2.0 Methods

### 2.1. Study design

Cochrane SR guidance was followed when performing our study selection and data extraction [19]. Our protocol is registered as a preprint on the Research Square server [20].

### 2.2 Search methods

#### 2.2.1 Database

As the basis for our search, we used an existing database of 1218 studies (2000–2020) collated from a bibliometric study [21]. The bibliometric study searched MEDLINE (Ovid), Epistemonikos, and the Cochrane Database of SRs of Interventions (CDSR) between January 1, 2000 and December 30, 2020. All studies included in the database: (a) synthesised the results of SRs, (b) systematically searched for evidence in a minimum of two databases, and (c) conducted their search using a combination of text words and MeSH terms. All included studies also had a full description of methods in the main body of the paper and focused on clinical or public health interventions.

Within this database, we identified Discordant Reviews using the EndNote search function and Boolean logic to identify the following words: overlap*[title/abstract] or discrepan*[title/abstract] or discord*[title/abstract] or concord*[title/abstract] or conflict*[title/abstract] or Jadad [abstract].

#### 2.2.2 Medline (Ovid) search January to April 2021

In addition, we completed a more recent search in the first quarter of 2021 in MEDLINE (Ovid) using the following search string: (“SRs”.tiab or “meta-analyses”.tiab) AND (overlap.tiab or discrepant.tiab or discordant.tiab or difference.tiab or conflicting.tiab or Jadad.ab). This search was conducted on April 18, 2021.

### 2.3 Screening

#### 2.3.1 Process for screening

Our screening form was piloted by all authors on 20 studies out of a possible 1251 identified by our searches to ensure high levels of agreement and common definitions of eligibility criteria. Articles were screened as full-text publications independently by two authors. Discrepancies were resolved by consensus, and arbitration by a third reviewer when necessary.

#### 2.3.2 Stage 1 screening criteria

We first screened the studies to include all those aiming to assess discordant results across SRs on similar clinical, public health or policy questions. Studies assessing discordance can assess (a) discordant results, or (b) discordant interpretations of the results and conclusions. Both studies examining (a) and (b) were eligible using any approach (e.g. [22–25]). We thus did not restrict our eligibility based on a study’s definition of discordance. Studies meeting stage 1 criteria continued onto stage 2 screening.

#### 2.3.3 Stage 2 screening criteria

In this stage, we selected studies that met the following inclusion criteria:

- Included a minimum of two SRs with a meta-analysis of RCTs, but may have included other study types beyond RCTs; and.
- Explicitly used the Jadad algorithm to choose between two or more discordant SRs for the primary outcome.

We included studies in any language and reviewers fluent in other languages used Google translate to aid in screening of non-English studies. Studies were eligible regardless of publication status and publication date.

### 2.4 Extraction of the primary intervention and outcome

As a systematic approach for assessing discordance, we first identified the primary outcome from each Discordant Review. We initially searched for the primary outcome as explicitly defined in the title, abstract, objectives, introduction, or methods sections [26, 27]. If the primary outcome was not identified in any of these sections, we deferred to the first outcome mentioned in the manuscript [26, 27].

The primary intervention was selected based on its relation to the primary outcome. If multiple interventions were assessed by the primary outcome, we chose the first intervention highlighted in the title or abstract [27]. We then determined which of the included SRs with meta-analysis of RCTs addressed the primary outcome and primary intervention.

The primary intervention and outcome were extracted by two authors independently, and any disagreement was discussed until consensus was reached.

### 2.5 Blinding of Jadad results in the discordant reviews

All included manuscripts underwent a blinding process where one reviewer independently deleted content related to Jadad results prior to our independent Jadad assessment. The one reviewer deleted pertinent components of the: abstract, highlights, results of the Jadad assessment, and discussion/conclusions sections, using Adobe Acrobat Pro or the freeware PDFCandy (https://pdfcandy.com). This individual was not involved in the subsequent Jadad assessment. Authors involved in the Jadad assessments were also instructed not to search for and read included Discordant Reviews prior to or during the assessment.

### 2.6 Achieving consensus instructions on how to do a Jadad assessment

The Jadad paper provides an algorithm for decision makers to choose across SRs and to identify sources of inconsistency and discordance, including differences in questions, inclusion/exclusion criteria, extracted data, methodological quality assessments, data combining, and statistical analysis methods. Within the Jadad manuscript there is little detailed guidance regarding the practical operationalisation of the algorithm. As such, out team met virtually to discuss interpretation and application of the algorithm, and to decide upon clear and specific decision rules for each Jadad algorithm step. Feedback was solicited and decision rules were adjusted until consensus was achieved. Our final interpretation of the Jadad algorithm steps and our decision rules are found in **Appendix A**, as well as in eight instructional videos located at: https://osf.io/2z7a5/.

### 2.7 Piloting Jadad assessments

The consensus instructions underwent pilot testing where further feedback was solicited and adjustments were made. Three Discordant Reviews [6, 26, 27] were used to pilot the Jadad assessments using our instructions. Two reviewers piloted three assessments using the Jadad algorithm independently and compared to a second reviewer’s assessment to identify discrepancies, which were resolved through discussion. Any necessary revisions and clarifications identified through this exercise were noted in our instructions (Sect. 2.8 and **Appendix A**).

### 2.8 Jadad assessment instructions

**Fig. 1:**
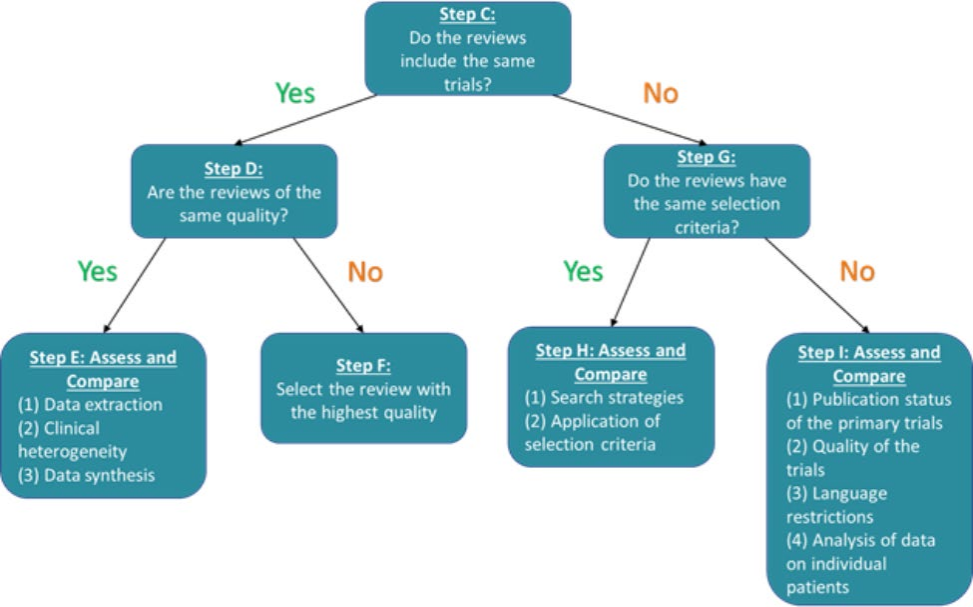
Jadad Algorithm (1997)

Briefly, Step A of the Jadad algorithm involves examining if the included SRs’ question match the Discordant Review’s question using a PICO framework [8]. If the clinical, public health, or policy questions were not identical, then Step B prompts a user to choose the SR closest to the decision makers’ question and no further assessment is necessary. If multiple SRs are found with the same PICO as the Discordant Review, then Step C should be investigated. As we were using Discordant Reviews with the same PICO as their included SRs, we started with Step C in the Jadad algorithm (Fig. 1).

**Step C** asks whether the same RCTs were included across the SRs (Fig. 1). If the SRs contained the same RCTs, then the assessor moved to **Step D** and assessed whether the SRs were of the same methodological quality or risk of bias. The AMSTAR (A MeaSurement Tool to Assess systematic Reviews) [22], the updated AMSTAR 2 [23] and outdated Oxman-Guyatt [24] are examples of tools used to assess methodological quality of SRs, and the ROBIS (Risk of Bias Assessment Tool for Systematic Reviews)( [25] tool is used to assess the risk of bias in SRs. In **Step D**, we either: (a) extracted the AMSTAR [22], AMSTAR 2 [23], or ROBIS [25] assessments from the Discordant Reviews, or if this was not done, (b) we conducted our own risk of bias assessment using the ROBIS tool [25]. If the included reviews varied in quality, the review of the highest quality was chosen at **Step F**.

If the SRs were of the same quality/risk of bias, then the next step is **Step E** - to assess and compare data extraction, clinical heterogeneity, and data synthesis across the reviews. Details about how we assessed this multi-tiered step is found in **Appendix A**.

If the SRs did not include the same trials, an assessment of the RCTs’ eligibility criteria as reported by the SRs was made at **Step G**. We found eligibility criteria information from the main text in the Discordant Reviews’ methods section, or in a table of characteristics. If the information was unavailable in the Discordant Review, two authors extracted the PICO eligibility criteria independently from the included SRs. Any discrepancies were resolved by discussion, and when necessary, with the involvement of a third reviewer.

If the SRs were determined to have sufficiently similar eligibility criteria, **Step H** prompts the assessor to compare the search strategies and the application of eligibility criteria across SRs to make a selection. If the eligibility criteria are not the same, **Step I** explores the publication status, quality, language, and availability of data on individual patients across the SRs. We applied multi-tiered hierarchical decision rules for these steps described in detail in **Appendix A**.

### 2.9 Study outcomes

#### 2.9.1 Comparing results from our Jadad assessment with the Discordant Review authors’ assessment

We replicated the Jadad assessments and evaluated whether we chose the same SR, whether a Cochrane SR was chosen, and whether we followed the same steps as the Discordant Review authors. We also evaluated the utility, efficiency, and comprehensiveness of the Jadad algorithm, and defined them as:

- Utility: Is the Jadad algorithm easy to use? (Sect. 2.9.2)
- Efficiency: How much time does it take to apply the Jadad algorithm?
- Comprehensiveness: Is the Jadad algorithm missing methods that might explain discordance (e.g., publication recency)?
- Reproducibility: What are the possible reasons for reproducibility or lack thereof?

Our timed Jadad assessments and ease of use ratings started after Steps C and G were completed. We therefore can only report our time and utility outcomes to do a partial Jadad assessment (Steps H and I). Three Discordant Reviews [6, 13, 26] were used to pilot the Jadad assessments, and were excluded from our assessment of the amount of time it took us to complete Steps H and I. We also compared our interpretation of how to use the Jadad algorithm with the Discordant Review authors’ interpretation.

#### 2.9.2 “Ease of use” outcome measure

The Jadad algorithm was assessed for ease of use by each assessor. A colour-coded ranking system was applied (green, yellow, red) based on how easy or difficult the assessment was judged to be for the user. The rating was based on the following rubric:

- The step can be accomplished easily by the reviewer, due to low cognitive load or because it’s a recognised method (green).
- The step requires a notable degree of cognitive load by the reviewer but can generally be accomplished with some effort (yellow).
- The step is difficult for the reviewer, due to significant cognitive load or confusion; some reviewers would likely fail or abandon the task at this point (red).

#### 2.9.3 ROBIS assessments

We assessed all SRs included in the Discordant Reviews for risk of bias using the ROBIS tool [25]. We chose to do this assessment (which is not part of Jadad) to gain knowledge about whether the Discordant Review authors or ourselves chose the SR which was at lowest risk of bias.

### 2.10 Data extraction

Information and data required to complete the Jadad algorithm were first sought directly from the Discordant Reviews, and if not reported, the full texts of the included SRs. The outcomes were extracted from 124 data items outlined in **Appendix B**. Discordant Review-level and SR-level data were extracted by two authors independently at full-text, and in the case when consensus was not reached, a third author arbitrated. Two reviewers also performed independent extractions of each Discordant Review’s interpretation of the Jadad algorithm steps. Any challenges or barriers that authors identified to using the Jadad algorithm were also extracted.

### 2.11 Data analysis

Our analyses were performed (a) descriptively for qualitative data, (b) using frequencies and percentages for categorical data, and (c) using median and interquartile range (IQR) for continuous data.

### 2.12 Deviations to our protocol

Due to the complexity of the Jadad instructions, we made several deviations to our protocol, which are outlined in **Appendix C**.

## 3.0 Results

### 3.1 Search results from the bibliometric study

**Fig. 2:**
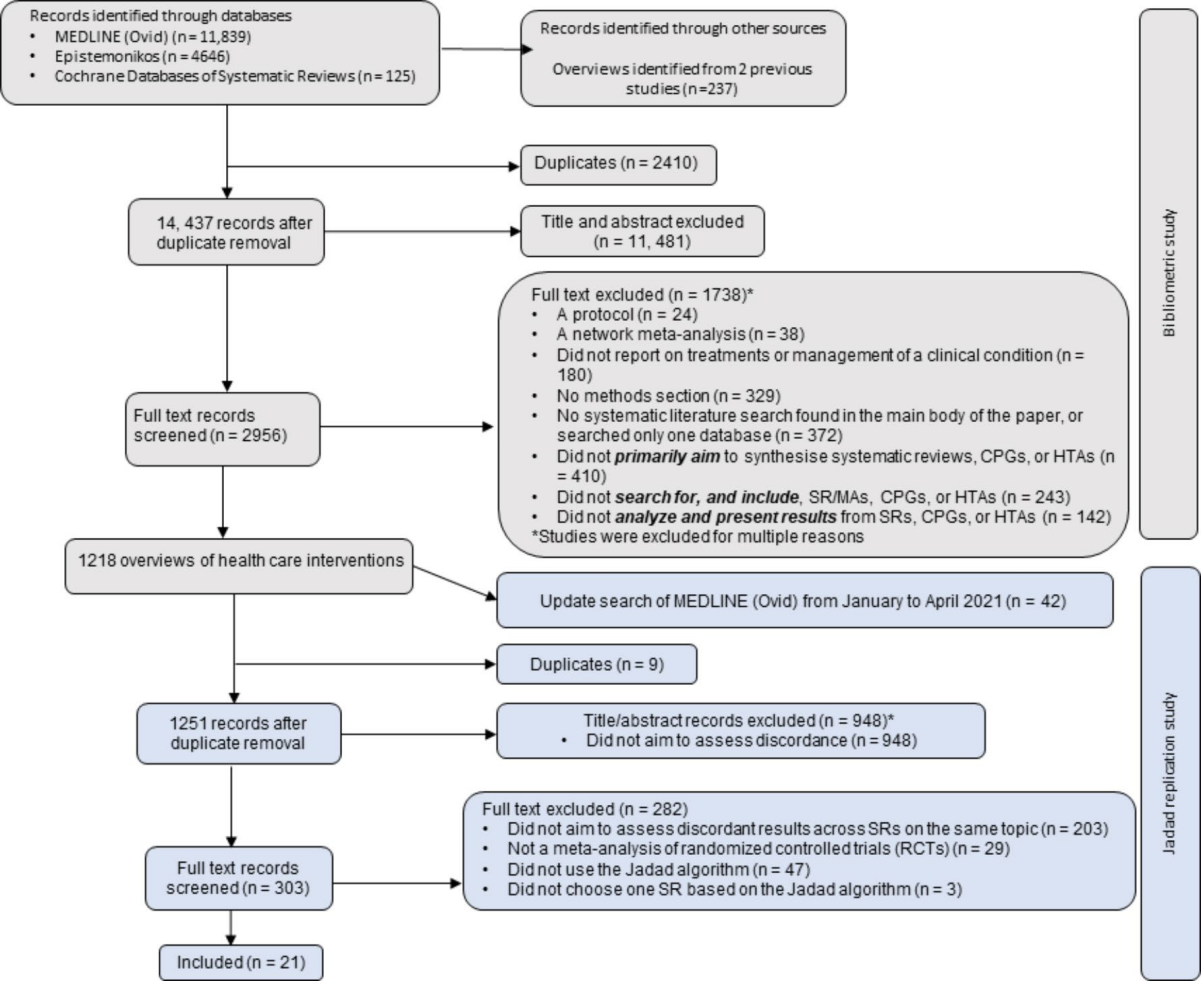
Study selection flowchart The first section in grey of the flowchart outlines the steps taken to select studies for the bibliometric study, and the second section in blue outlines our selection of studies for the Jadad replication.

We retrieved 16,610 records from the MEDLINE (Ovid), CDSR, and Epistemonikos databases, and 237 records from other sources (Fig. 2). Of the remaining 14,437 records after removal of duplicates, 11,481 were excluded at the title/abstract stage, and 1738 were excluded at the full text stage. A total of 1,218 articles were included that met our eligibility criteria.

### 3.2 Search results from the Jadad replication study

We updated our search in April 2021, which yielded 1,251 records of which 948 were excluded at the title/abstract stage. We screened 303 full text records, and of these, 24 studies included at least two SRs with meta-analysis of RCTs and used the Jadad algorithm. However, after scrutiny, we excluded another three studies [27–29] from our analysis as they did not choose one or multiple SRs based on the Jadad algorithm and did not follow the Jadad steps. These studies are described separately in **Appendix D**.

### 3.2 Characteristics of discordant reviews

The most common nomenclature for this study type was a ‘SR of overlapping meta-analyses’, or a ‘SR of discrepant meta-analyses’. One study was described by the authors as a ‘Systematic review of systematic reviews’ in the title and their primary aim was to assess discordance across the SRs, not to synthesize the results of multiple SRs. Other studies self-identified as SRs in the title but they did not collect and analyse primary study data. Instead, the authors of these articles assessed discordance across SRs. Despite the variety in terminology across our included studies, we have called them Discordant Reviews to distinguish them from their constituent SRs.

The 21 Discordant Reviews using the Jadad algorithm were published between 2014 and 2020 (Table 1), except for Poolman 2007 [30]. Overall, more than half of the 21 Discordant Reviews were from China alone; and when counted with the USA, accounted for most of the included Discordant Reviews. Eighteen of the 21 studies (86%) were done on conditions of the bone and joint, with the rest being on cholecystitis, orthodontically induced white spot lesions, and non-small cell lung cancer.

Within each Discordant Review, the number of included SRs with meta-analysis ranged from 2 to 7, except for Xing 2016, which included 10. The number of authors of the Discordant Reviews ranged from 2 to 10.

**Table 1:** Characteristics of included Discordant Reviews (n = 21)

First Author Year	Objective	Primary outcome	Primary intervention	Country of corresponding author	Health area addressed (ICD-10 Medical Classification)	# Authors per Discordant Review	# Systematic reviews with meta-analysis of RCTs	Discordant Review authors’ conclusion
**Bakdach 2020** [13]	Appraise evidence on the management of orthodontically induced white spot lesions (OIWSLs) and choose the best evidence	Incidence of lesions	Topical fluoride toothpaste and/or brush on gel [varnish], or foam	Syria	Caries limited to enamel (K02.0)	2	3	“Topical fluorides yielded a 25–30% prevention of OIWSLs; however, their effect on reversing OIWSLs is unclear.”
**Chalmers 2015** [26]	Critically evaluate meta-analyses for arthroscopic versus open stabilization techniques for shoulder instability	Recurrent instability	Open versus arthroscopic shoulder stabilization	USA	Other instability of joint (M25.3)	7	2	“There are no significant differences in failure rates “[i.e. recurrent instability].
**Bolland 2014** [6]	Explore why discordant results arise across meta-analyses on vitamin D supplements and fracture	Hip fracture	Vitamin D (+/- Calcium)	New Zealand	Fracture of unspecified body region (T14.2)	2	4	“Each of the 3 meta-analyses concluded that vitamin D alone does not prevent fractures, regardless of dose.”
**Grassi 2018** [31]	Assess and analyze current evidence regarding patellar resurfacing and non-resurfacing in TKA	Risk of reoperation	Patellar resurfacing versus non-resurfacing in total knee arthroplasty	Italy	Unspecified complication of internal orthopaedic prosthetic device, implant and graft (T84.9)	8	5	“Risk of re-operation was higher after non-resurfacing, however, when the authors considered only high quality RCTs, no differences were reported.”
**Erickson 2015** [32]	Compare nonoperative and operative treatment of patellar dislocations to determine the best available evidence	Recurrent patellar dislocations	Nonoperative versus operative treatment	USA	Recurrent dislocation of patella (M22.0)	8	2	“Operative treatment of acute patellar dislocations may result in a lower rate of recurrent dislocations than nonoperative treatment.”
**Chen P 2019** [15]	Determine which meta-analysis provides the best available evidence for the use of PRP in the treatment of knee osteoarthritis (KOA) patients	Pain as measured by the WOMAC total score	Platelet-rich plasma (PRP) Injection versus hyaluronic acid (HA) injection or placebo	China	Gonarthrosis, unspecified (M17.9)	8	4	“Intra-articular PRP injection is more effective in terms of pain relief and function improvement in the treatment of KOA patients than HA and placebo”
**Chen X 2018** [33]	Identify the benefits and disadvantages of unilateral PKP versus bilateral PKP as found in numerous discordant meta-analyses	Pain as measured by the short-term VAS score	Unilateral percutaneous balloon kyphoplasty (PKP) versus bilateral PKP	China	Unspecified osteoporosis with pathological fracture (M80.9)	10	5	“Unilateral PKP required shorter surgical time and less cement volume, offering better pain relief and quality of life at post-operative short-term follow-ups.”.
**Xu 2017** [34]	Interpret and select amongst discordant MAs and provide surgical recommendations for displaced midshaft clavicle fracture	Fracture non-union	Intramedullary fixation (IF) versus plate fixation (PF) for displaced midshaft clavicle fracture	China	Fracture of clavicle (S42.0)	7	5	“The best available evidence indicated that the differences between IF and PF were not significant in terms of shoulder function or the rate of treatment failure [includes fracture union]”
**Song 2016** [35]	Assess discordant MAs for treating acute cholecystitis and timing of laparoscopic cholecystectomy	Bile duct injury	Early (within 7 days of the onset of symptoms) versus delayed laparoscopic cholecystectomy for acute cholecystitis	China	Acute cholecystitis (K81.0)	6	6	“The best available evidence indicated a nonsignificant difference in bile duct injury.”
**Zhao 2015** [18]	Compare surgical and conservative interventions for the treatment of displaced midshaft clavicular fractures	Function as assessed by the constant score	Surgical versus conservative treatment	China	Fracture of clavicle (S42.0)	3	3	“Surgical treatment provides a lower rate of overall treatment failure and a better functional outcome, but is associated with more implant-related complications.”
**Poolman 2007** [30]	Evaluate reasons for differences in systematic reviews on bone-patellar tendon-bone or hamstring tendon autograft	Knee stability as measured by pivot shift test	Hamstring autograft versus bone-patellar tendon-bone autograft	Canada	Sprain and strain involving (anterior)(posterior) cruciate ligament of knee (S83.5)	4	3	“The currently available best evidence suggests that hamstring tendon autografts are superior for preventing anterior knee pain, and there is limited evidence that bone-patellar tendon-bone autografts provide better stability.”
**Mascarenhas 2014** [17]	Determine whether double-row (DR) or single-row (SR) rotator cuff repair provides superior clinical outcomes and structural healing	Function as measured by the constant score	Single row (SR) versus double row (DR) rotator cuff repair (RCR) techniques	USA	Rotator cuff syndrome (M75.1)	7	3	“It was determined that, according to the current best available evidence, DR RCR provides superior patient outcomes and structural healing when compared with SR RCR.”
**Tan 2018** [36]	Compare clinical safety and efficacy of unilateral versus bilateral PKP for treating osteoporotic vertebral compression fracture (OVCF)	Pain as measured by the short-term VAS score	Unilateral versus bilateral percutaneous balloon	China	Unspecified osteoporosis with pathological fracture (M80.9)	6	6	“Compared with bilateral PKP, unilateral PKP produced a shorter surgery time, smaller dosage of cement, lower risk of cement leakage, and relieved a higher degree of intractable pain at short-term follow-up after surgery.”
**Xing 2016** [37]	Perform a systematic review of overlapping meta-analyses investigating the efficacy and safety of HA for KOA	Early and late knee pain	HA versus placebo	China	Gonarthrosis, unspecified (M17.9)	7	10	“HA is an effective intervention in treating KOA without increased risk of adverse events.”
**Mascarenhas 2015** [38]	Compare double-bundle (DB) or single-bundle (SB) anterior cruciate ligament reconstruction (ACL-R)	Knee stability measurements by pivot-shift testing	SB versus DB anterior cruciate ligament reconstruction	USA	Sprain and strain involving (anterior)(posterior) cruciate ligament of knee (S83.5)	7	6	“The current best available evidence suggests that DB ACL-R provides better postoperative knee stability than SB ACL-R, whereas clinical outcomes and risk of graft failure are similar between techniques.”
**Houck 2017** [39]	Compare early versus delayed motion rehabilitation protocols after rotator cuff repair to determine which MAs provide the best available evidence.	Range of motion (general ROM; forward flexion; external rotation)	Early versus delayed motion rehabilitation protocols	USA	Rotator cuff syndrome (M75.1)	5	5	“The current, best available evidence suggests that early motion improves ROM after rotator cuff repair but increases the risk of rotator cuff retear.”
**Pekala 2019** [40]	Present a comprehensive review based on the most up-to-date MAs on the association of FokI with IDD	Intervertebral disc degeneration (IDD)	FokI (rs2228570) polymorphism	Poland	Intervertebral disc disorder, unspecified (M51.9)	7	7	“Based on the results from studies published to date, there is no evidence of an association between the FokI polymorphism and IDD in the general population.”
**Zhiyong 2019** [41]	Select the best evidence between unilateral and bilateral balloon kyphoplasty for osteoporotic vertebral compression fractures (OVCFs)	Pain as measured by the short term VAS scores	Unilateral versus bilateral balloon kyphoplasty	China	Unspecified osteoporosis with pathological fracture (M80.9)	5	6	“Unilateral kyphoplasty is more advantageous, effective and safe, compared to bilateral kyphoplasty for the treatment of OVCFs.”
**Fu 2019** [42]	Provide recommendations for displaced 3-part and 4-part fractures of proximal humerus based on the best evidence	Function as assessed by the constant score	Surgical versus non-surgical treatment	China	Fracture of upper end of humerus (S42.2)	4	4	“No statistically significant differences were found in the constant score between surgical and non-surgical treatments.”
**Zhao 2015** [43]	Compare intramedullary nail and plate fixation for the treatment of humeral shaft fractures	Non-union	Intramedullary nail versus plate fixation	China	Fracture of shaft of humerus (S42.3)	4	4	“The differences between intramedullary nail and plate fixation were not significant in fracture union.”
**Guo 2018** [44]	Offer treatment recommendations based on current best evidence of Shenyi Capsule plus chemo versus chemo of non-small cell lung cancer	Disease control rate	Shenyi Capsule plus chemo versus chemo alone	China	Malignant neoplasm of unspecified part of unspecified bronchus or lung (C34.90)	5	4	“Shenyi capsule plus chemo could increase incidence of short-term efficacy, improve the quality of life and survival rate in comparison to chemotherapy.”

ACL-R: Anterior cruciate ligament reconstruction; DB: Double-bundle; DR: Double-row; HA: hyaluronic acid; IDD: Intervertebral disc degeneration; IF: Intramedullary fixation; KOA: knee osteoarthritis; MA: meta-analysis; OIWSLs: orthodontically induced white spot lesions; OVCF: Osteoporotic vertebral compression fracture; PKP: Percutaneous balloon kyphoplasty; PF: Plate fixation; PRP: platelet-rich plasma; RCR: rotator cuff repair; ROM: Range of motion; SB: Single-bundle; SR: Single-row

### 3.3 Replication of Jadad assessments and Jadad steps assessed

Over the 21 Jadad assessments we conducted, we did not once answer yes to Step C, meaning the SRs included in the 21 Discordant Reviews did not contain the same RCTs.

As all SRs across the 21 Discordant Reviews contained different RCTs, we then progressed to assess Step G (i.e. do SRs contain the same selection criterion?). Of these, 17 Discordant Reviews (81%) were determined to have the same selection criteria across their included SRs so they moved onto to Step H, and four Discordant Reviews (19%) did not so they moved onto Step I.

Since the included SRs did not contain the same RCTs, we did not use Step D, E and F as final decision steps to select a SR. Within Step I of the algorithm, we found that no SRs performed an individual patient meta-analysis, and this sub-step was not used in assessing discordance.

#### 3.3.1 Frequency of agreement and disagreement in the selection of the most appropriate systematic review(s) using the Jadad algorithm between ourselves and the Discordant Review authors

Of the 21 Jadad assessments, eight decisions (38%) on which SR(s) to choose agreed, and 13 (62%) disagreed (Tables 2 and 3). The author groups of 18 Discordant Reviews chose one SR, two groups chose two SRs, and one group chose three SRs. Over 21 Jadad assessments, we chose one SR in 16 instances, two SRs on four occasions, and three SRs once. In four cases, we chose the same SR as the Discordant Review authors, but also chose one additional SR.

Of the 21 Jadad assessments, 19 (90%) reported the Jadad step they used to make their final SR selection (hereafter called the “final decision step”). Of the 13 Jadad assessments that disagreed, in six instances we used the same final decision step as the Discordant Review authors, and we chose a different step seven times. Of the eight Jadad assessments between ourselves and the Discordant Review authors that agreed, six reported the Jadad final decision step. Of these, we used the same step to make our decision three out of six times.

**Table 2:** Agreement and disagreement in choice of systematic review(s) in replicated Jadad assessments

First Author Year	Jadad assessment primary outcome	Jadad assessment primary intervention	# MAs of RCTs	Discordant Review authors’ or our Jadad assessments	Jadad final decision step	SR(s) chosen	Tool used by the Discordant Review authors to assess the quality of SRs (judgment if AMSTAR used)	Cochrane or non-Cochrane SR	ROBIS assessment
**Bakdach 2020**	Incidence of white spot lesions	Topical fluoride toothpaste, brush on gel and/or foam	3	Discordant Review authors	Step I	Sardana 2018	AMSTAR-2 (Moderate quality)	Non-Cochrane	Low risk
				Our Choice	Step I	Tasios 2019 (and Sardana 2019)	AMSTAR-2 (Critically low quality)	Non-Cochrane	Low risk
**Chalmers 2015**	Rate of recurrence	Arthroscopic surgery	2	Discordant Review authors	Step I	Pulavarti 2007	Oxman-Guyatt	Cochrane	Low risk
				Our Choice	Step H	Lenters 2007 (and Pulavarti 2007)		Non-Cochrane	Low risk
**Bolland 2014**	Hip fracture	Vitamin D +/- calcium versus placebo	4	Discordant Review authors	NR	Avenell 2009	AMSTAR (High quality)	Cochrane	High risk
				Our Choice	Step H	Avenell 2009			
**Grassi 2018**	Re-operation rate	Patellar resurfacing versus non-resurfacing	5	Discordant Review authors	Step I	He 2011	AMSTAR (High quality)	Non-Cochrane	Low risk
				Our Choice	Step H	He 2011			
**Erikson 2015**	Patellar instability	Operative versus non-operative treatment	2	Discordant Review authors	NR	Hing 2011	Oxman-Guyatt	Cochrane	Low risk
				Our Choice	Step H	Hing 2011			
**Chen 2019**	Pain using WOMAC total score	PRP injection	4	Discordant Review authors	Step I	Shen 2017	Oxman-Guyatt	Non-Cochrane	Low risk
				Our Choice	Step H	Dai et al. 2017		Non-Cochrane	Low risk
**Song 2016**	Bile duct injury	Early versus delayed laparoscopic cholecystectomy	6	Discordant Review authors	Step H	Cao 2015	AMSTAR (High quality)	Non-Cochrane	High risk
				Discordant Review authors	Step H	Wu 2015	(High quality)	Non-Cochrane	Low risk
				Our Choice	Step H	Gurusamy 2013	(Highest quality)	**Cochrane**	Low risk
**Chen 2018**	Short term VAS	Unilateral PKP versus bilateral PKP	5	Discordant Review authors	Step I	Feng 2015	AMSTAR (High quality)	Non-Cochrane	High risk
				Our Choice	Step I	Feng 2015	(High quality)		
**Xu 2017**	Non-union	Intramedullary fixation versus plate fixation	5	Discordant Review authors	Step H	Lenza 2015 and Hussain 2016	AMSTAR (Highest and High quality)	Cochrane and Non-Cochrane	Low and High
				Our Choice	Step H	Lenza 2015 and Hussain 2016	(Highest and High quality)		Low and High risk
**Zhao 2015a**	Constant score	Surgical versus conservative treatment	3	Discordant Review authors	Step H	Lenza 2013	AMSTAR (High quality)	Cochrane	Low risk
				Our Choice	Step H	Lenza 2013	(High quality)		
**Tan 2018**	Short-term VAS score	Unilateral versus bilateral percutaneous balloon	6	Discordant Review authors	Step H	Feng 2015	AMSTAR (High quality)	Non-Cochrane	Low risk
				Our Choice	Step H	Lin 2013	(High quality)	Non-Cochrane	High risk
**Poolman 2007**	Stability	Hamstring versus bone-patellar tendon-bone autograft	3	Discordant Review authors	Step F	Biau 2006	Oxman-Guyatt	Non-Cochrane	Low risk
				Our Choice	Step H	Biau 2006			
**Mascarenhas 2014**	Constant score	Single versus double row rotator cuff repair	3	Discordant Review authors	Step I	Millett 2014	Oxman-Guyatt	Non-Cochrane	Low risk
				Our Choice	Step H	Sheibani-Rad 2013		Non-Cochrane	High risk
**Xing 2016**	Early and late knee pain	Hyaluronic acid versus placebo	10	Discordant Review authors	Step I	Bellamy 2006	AMSTAR (Highest quality)	Cochrane	High risk
				Our Choice	Step H	Richette 2015 (and Bellamy 2006)	(High quality)	Non-Cochrane	High risk
**Mascarenas 2015**	Pivot-shift test score	Single row versus double row rotator cuff repair techniques	6	Discordant Review authors	Step I	Li 2014, van Eck 2012 and Tiamklang 2012	Oxman-Guyatt	Non-Cochrane, Cochrane, and Non-Cochrane	Low, Low and Low risk
				Our Choice	Step H	Li 2014, van Eck 2012 and Tiamklang 2012			
**Guo 2018**	Disease control	Shenyi capsule and chemo versus chemo alone	4	Discordant Review authors	Step I	Xia 2014	AMSTAR (Moderate quality)	Non-Cochrane	High risk
				Our Choice	Step H	Hu 2011	(Low quality)	Non-Cochrane	Low risk
**Houck 2017**	Range of motion	Early versus delayed motion rehabilitation	5	Discordant Review authors	Step I	Riboh 2014	Oxman-Guyatt	Non-Cochrane	Low risk
				Our Choice	Step H	Chan 2014		Non-Cochrane	High risk
**Pekala 2019**	Inter-vertebral disc degeneration	Fokl polymorphism	7	Discordant Review authors	Step I	Pabalan 2016	AMSTAR (Moderate quality)	Non-Cochrane	High risk
				Our Choice	Step I	Nong 2016 (and Pabalan 2016)	(Moderate quality)	Non-Cochrane	Low risk
**Zhiyong 2019**	Short term VAS scores	Unilateral versus bilateral balloon kyphoplasty	6	Discordant Review authors	Step I	Sun et al. 2016	AMSTAR (Moderate quality)	Non-Cochrane	Low risk
				Our Choice	Step I	Feng 2015	(Moderate quality)	Non-Cochrane	Low risk
**Fu 2019**	Function	Surgical versus non-surgical treatment	4	Discordant Review authors	Step H	Rabi et al. 2015	AMSTAR (High quality)	Non-Cochrane	High risk
				Our Choice	Step H	Handoll 2012	(Highest quality)	Cochrane	Low risk
**Zhao 2015b**	Non-union	Plate fixation	4	Discordant Review authors	Step I	Ouyang 2013	AMSTAR (Moderate quality)	Non-Cochrane	Low risk
				Our Choice	Step H	Heineman 2010	(Moderate quality)	Non-Cochrane	Low risk

AMSTAR: A MeaSurement Tool to Assess systematic Reviews; Oxman-Guyatt: Oxman-Guyatt quality assessment questionnaire; PKP: Percutaneous balloon kyphoplasty; PRP: platelet-rich plasma; ROBIS: Risk of Bias Assessment Tool for Systematic Reviews

#### 3.3.2 Frequency of agreement and disagreement in systematic review findings

Ten out of 13 (77%) discordant Jadad assessments led to agreement in the findings (direction of effect was the same) (Table 3). The remaining three independent Jadad assessments that disagreed led to a different direction of the effect estimates.

Overall, 18 out of the 21 (86%) independent Jadad assessments agreed in direction of the findings despite 13 having chosen a different SR. We present a case study in **Appendix E** to illustrate the clinical impact of choosing one SR using the Jadad algorithm.

#### 3.3.3 Cochrane versus non-cochrane reviews chosen

Of the 21 Jadad assessments, four Cochrane reviews were chosen either by us or the Discordant Review authors (Table 3). On four occasions we chose a Cochrane review, and twice the Discordant Review authors chose a Cochrane review.

**Table 3:** Agreement and disagreement in systematic review findings from replicated Jadad assessments

Discordant Review	Discordant Review authors’ or our Jadad assessments	Review(s) chosen by the Discordant Review (First author Year)	Cochrane or non-Cochrane SR	Risk of bias in the review	Type of effect estimate	Pooled effect sizes and 95% CI	Statistical significance (*p-value* of the effect estimate)	Results favourable, null, or unfavourable	Direction of effect
Bakdach 2020	Discordant Review authors	Sardana 2018	Non-Cochrane	Low	Risk ratio	0.39 (0.26–0.59)	0.005	Favourable	**Disagree**
	Our Choice	Tasios 2019 (and Sardana 2019)	Non-Cochrane	Low	Risk ratio	0.46 (0.18-1.15)	0.1	Null	
Chalmers 2015	Discordant Review authors	Pulavarti 2007	**Cochrane**	Low	Risk ratio	0.89 (0.09, 8.72)	0.92	Null	Agree
	Our Choice	Lenters (and Pulavarti 2007)	Non-Cochrane	Low	Risk ratio	1.31 (0.51, 3.34)	0.58	Null	
Chen 2019	Discordant Review authors	Shen 2017	Non-Cochrane	Low	Mean difference	-17.39 (-22.32, -12.46)	< 0.00001	Favourable	Agree
	Our Choice	Dai et al. 2017	Non-Cochrane	Low	Mean difference	-2.83 (-4.26, -1.39)	0.0001	Favourable	
Fu 2019	Discordant Review authors	Rabi et al. 2015	Non-Cochrane	High	Mean difference	1.63 (-2.84, 6.11)	0.47	Null	Agree
	Our Choice	Handoll 2012	**Cochrane**	Low	Mean difference	2.36 (-3.52, 8.24)	0.43	Null	
Guo 2018	Discordant Review authors	Xia 2014	Non-Cochrane	High	Risk ratio	1.19 (1.05, 1.35)	0.006	Favourable	Agree
	Our Choice	Hu 2011	Non-Cochrane	Low	Odds Ratio	3.34 (1.92, 5.81)	< 0.0001	Favourable	
Houck 2017	Discordant Review authors	Riboh 2014	Non-Cochrane	Low	Mean difference	14.70 (5.52, 23.87)	0.002	Favourable	Agree
	Our Choice	Chan 2014	Non-Cochrane	High	Mean difference	1.05 (0.03, 2.06)	0.04	Favourable	
Mascarenhas 2014	Discordant Review authors	Millett 2014	Non-Cochrane	Low	Mean difference	-3.7 (-8.8, 1.4)	0.16	Null	Agree
	Our Choice	Sheibani-Rad 2013	Non-Cochrane	High	Mean difference	0.159 (-0.08, 0.40)	0.255	Null	
Pekala 2019	Discordant Review authors	Pabalan 2016	Non-Cochrane	High	Odds ratio	0.99 (0.75, 1.31)	0.95	Null	Agree
	Our Choice	Nong 2016 (and Pabalan 2016)	Non-Cochrane	Low	Odds ratio	1.13 (0.76–1.69)	0.55	Null	
Song 2016	Discordant Review authors	Cao 2015	Non-Cochrane	High	Risk ratio	0.41 (0.07, 2.52)	0.34	Null	Agree
	Discordant Review authors	Wu 2015	Non-Cochrane	Low	Risk ratio	0.98 (0.2, 4.75)	0.98	Null	
	Our Choice	Gurusamy 2013	**Cochrane**	Low	Odds ratio	0.49 (0.05, 4.72)	0.54	Null	
Tan 2018	Discordant Review authors	Feng 2015	Non-Cochrane	Low	Mean difference	-0.18 (-0.36, -0.00)	0.04	Favourable	**Disagree**
	Our Choice	Lin 2013	Non-Cochrane	High	Mean difference	0.05 (-0.49, 0.59)	0.87	Null	
Xing 2016	Discordant Review authors	Bellamy 2006	**Cochrane**	High	Mean difference	-13.00 (-17.77, -8.33)	< 0.00001	Favourable	Agree
	Our Choice	Richette 2015 (and Bellamy 2006)	Non-Cochrane	High	Standardised mean difference	-0.21 (-0.32, -0.1)	NR	Favourable	
Zhao 2015a	Discordant Review authors	Ouyang 2013	Non-Cochrane	Low	Risk ratio	1.20 (0.63, 2.28)	0.58	Null	Agree
	Our Choice	Heineman 2010	Non-Cochrane	Low	Risk Ratio	0.71 (0.28, 1.76)	0.45	Null	
Zhiyong 2019	Discordant Review authors	Sun et al. 2016	Non-Cochrane	Low	Mean difference	-0.12 (-0.33, 0.09)	0.28	Null	**Disagree**
	Our Choice	Feng 2015	Non-Cochrane	Low	Mean difference	-0.18 (-0.36, -0.00)	0.04	Favourable	

#### 3.3.4 Interpretation of Jadad steps by the discordant review author

Overall, there were major differences in the interpretation of the Jadad algorithm across Discordant Review author groups. Several Discordant Reviews (n = 12) did not implement the Jadad algorithm in sequential steps but rather, used components of the algorithm to assess SRs. In addition, various factors outside the Jadad algorithm were assessed by half (n = 10/21) Discordant Review authors, including: databases that were searched, the GRADE (Grading of Recommendations, Assessment, Development and Evaluations) approach, randomization method, methods used to measure outcomes, measures used to explore heterogeneity, measures used for establishing comparative superiority or inferiority, statistical approaches used in analyses, presence of subgroup analyses, software used to perform the analyses, and sources funding.

Most Discordant Reviews (n = 12) used Step I as the final decision step of the algorithm. We interpreted Step I to consist of (I1) publication status, (I2) quality of RCTs, (I3) language restrictions, and (I4) analysis of individual patient data (IPD). We operationalised publication status to be whether SRs included both published and unpublished (grey literature). In contrast, several Discordant Review authors interpreted this step to consider the date or recency of the review (n = 5) or to account for only published literature (n = 3).

The second most common final decision step was Step H (n = 5). We interpreted Step H to consist of (H1) search strategies across SRs and (H2) inclusion criteria and duplicate independent screening of RCTs. Different Interpretations of H1 included: whether the SR was published in a medical journal, and which electronic databases were searched. Interpretations of H2 included: whether the SR reported publication status and language. Many Discordant Review authors were not clear in the details of how they interpreted this step. Some Discordant Review authors reported Step H as the final decision step but ignored the criteria for Step G and selected the SR with the highest number of RCTs (n = 2). The rationale behind this decision was not reported.

#### 3.3.4 Time and ease of use in completing steps H and I of the Jadad assessments

##### 3.3.4.1 Time to do steps H and I of the Jadad algorithm

Of the 18 Jadad algorithm assessments completed for Steps H and I, the average time was 60 min per review (**Appendix F and G**, Table 1). Ten Jadad assessments took between 15 and 47.5 min with an average of 4.3 SRs to assess (range 3–6). Nine out of the ten were rated as easy to assess, and one was rated as being moderately difficult to assess. These nine easy-rated assessments had evaluated Step H of the Jadad algorithm as the final decision step. The moderately rated assessment had evaluated Step I as the final decision step.

##### 3.3.4.1 Ease of use rating for final decision steps H and I of the Jadad algorithm

Of the 18 Jadad assessments we completed with final decision for Steps H and Step I, the median ease rating was easy (**Appendix F and G**, Table 1). Ten out of 18 (56%) assessments were rated easy, six (33%) were rated moderate, one moderate/hard (6%), and one hard (6%). All the assessments rated as easy were based on completing Step H. Of the easy assessments, the average number of SRs to assess was 4.4 (range 3–6 SRs). Of the eight moderate to hard assessments, three required assessment of Step I, and five Step H. They averaged 5.5 SRs to assess (range 2–10 SRs). By observation, we noticed that an easy Jadad assessment involved good reporting by the Discordant Review authors and the SR authors, the step assessed, and whether the Discordant Review interpreted the Jadad algorithm in a similar way than to us. By observation, we noticed that moderate to hard assessments involved inadequate reporting by the Discordant Review authors and discrepant data reported in their included SRs, having to conduct ROBIS assessments as the Discordant Review did not assess the quality of the SRs, and a greater number of SRs included.

#### 3.3.6 Comprehensiveness of the Jadad algorithm (gaps or completeness)

We identified several missing methods for explaining discordance. The algorithm did not account for the date of last literature search, nor did it account for publication recency. The number of primary studies included in the SRs was also not considered within the Jadad algorithm. Lastly, the certainty of evidence, as measured by the GRADE or other approaches, was not examined by the algorithm.

### 3.4 ROBIS assessments

We assessed 98 SRs which were included in our 21 Discordant Reviews using the ROBIS tool. A total of 41 SRs were at low risk of bias, and 57 SRs were at high risk of bias. From the 21 Discordant Reviews, we chose 19 low risk SRs and 9 high risk SRs, while the authors chose 17 low risk SRs and 8 high risk SRs. A more detailed assessment as well as the full ROBIS assessments for each SR are found in **Appendix H**. Our ROBIS judgments of high or low risk of bias for each SR are found in Table 2.

## 4.0 Discussion

### 4.1 Summary and interpretation of the most important results

In our investigation, we identified research examining discordance across comparable SRs using the Jadad algorithm [8] and attempted to replicate their findings. In 62% of cases, we were unable to replicate the findings and ultimately chose a different “best evidence” SR. The lack of guidance on how to operationalise the Jadad algorithm likely contributed to the different interpretations, and ultimately disagreement between our choice and the Discordant Review authors’ choice of SR. Several Discordant Reviews did not implement the Jadad algorithm in sequential steps which also may have led to us choosing a different review using the Jadad algorithm. By observation, whenever a Cochrane SR was included in a Discordant Review, the authors or us chose the Cochrane review as the best evidence. Overall, the raters assessed the Jadad algorithm as easy to use, taking average time was 60 min with an average of 4.3 SRs to partially assess. By observation, we noticed that Jadad assessments took a longer time when there was: (a) greater number of SRs to assess, (b) having to do a quality assessment for the included SRs (as this was missing from the Discordant Review), and (c) inadequate reporting by the Discordant Review authors.

Due to limited reporting, it was challenging to replicate or obtain a comprehensive understanding of Discordant Review authors’ use of the Jadad algorithm. Inadequate reporting on how the authors interpreted the Jadad algorithm and operationalised each step led to challenges in extracting the required information. Often, Discordant Review authors only discussed the final decision step of the algorithm, and did not discuss the other steps taken that led to the final decision.

The Jadad algorithm has several limitations in terms of comprehensiveness. The algorithm does not account for the date of last literature search, publication recency, the number of RCTs included in the SRs and certainty of evidence assessment (e.g. using the GRADE approach). Moreover across 21 assessments, quality/risk of bias at the SRs was not assessed.

The exponential growth of SRs means duplication and redundant reviews will become a greater problem for clinicians and policy makers. As there is no similar tool to assess discordance, the Jadad algorithm will continue to be used by researchers , and is indeed still being used today [13–15]. However, the algorithm is out of date, and therefore fails to incorporate advances in biases, methodological and statistical approaches to evidence synthesis [45, 46]. Major methodological advances published after its emergence include the PRISMA reporting standard (2009 and updated in 2020; [47, 48]), AMSTAR (2007 and update in 2017; [22, 23]), ROBIS (2018; [25]), and GRADE (2009; [49]). Statistical advances of current interest include multiple imputations to model missing data, meta-regression and model selection, living systematic reviews, and network meta-analyses [45, 46], to name a few.

Intuitively, SR quality should be an important consideration when comparing reviews, but this was never considered by our team or the authors of the 21 included Discordant Reviews because the entire left side of the Jadad algorithm (Steps D, E and F) was never applied. This should be considered a design flaw in the algorithm. SR quality was only considered in the algorithm when SRs evaluated the same RCTs, which is highly unlikely with the exponential growth in research output [1] and the difficulty in locating trials. In our sample, it never happened. When two (or more) SRs asked clinical, public health, or policy questions with similar eligibility criteria it would be logical to evaluate SR quality using validated tools like AMSTAR-2 or risk of bias tools such as ROBIS and either choose the highest quality SR or eliminate lower quality SRs when there are several to choose from.

### 4.2 Comparison of our study with other similar studies

No methodological investigations or replications of the Jadad algorithm were identified, and therefore we are not aware of any study to which we could directly compare our study results. We are only aware of studies that applied the Jadad algorithm for choosing the best SR. This is surprising, as it is well-known that in most cases there are several SRs available for a given question [3], and general rules for practitioners have been suggested on how to choose the best review [50]. Others have noted that unexplained discordance can also result in formulating the need to conduct a new review [11, 51]. This underpins the urgent need for further empirical investigations.

### 4.3 Implications when clinicians encounter multiple discordant systematic reviews on the same question

Evidence-based medicine is commonly defined as the conscientious, explicit, and judicious use of current best evidence in the process of decision-making related to patient care [52]. Medical knowledge grows every day, so that evidence is rapidly evolving, and it seems impossible to stay current [1]. For example, it is recommended that a general practitioner read 19 articles every day [53], and dedicate an average of one hour per week to keep abreast of the literature [53]. With the additional problem of conflicting results in seemingly identical research, clinicians may struggle to find the time and guidance on how to do this.

Without the help of an updated algorithm to assess discordant results across SRs, clinicians will have difficulty in identifying and choosing the best evidence and thus engaging in decision-making with their patients and clinical teams. Uncertainty, disagreements, and differences in SR results undermine the ability of a healthcare provider to make an informed clinical decision [29]. As an example of the clinical impact of discordant results of SRs [29], patient reimbursement for hyaluronic acid treatment was stopped because of some negative meta-analyses results [54, 55], despite the fact that other SRs [56, 57] cited beneficial effects, as did RCTs for certain preparations [58, 59].

All Discordant Reviews using Jadad in our sample address a focused clinical question (e.g. comparing only two interventions for a specific condition and population). These Discordant reviews chose one or a small subset of SRs which may bring about simplicity in terms of summarising the SR results (since there will only be one or a few SRs included), but may lead to a loss of potentially important information through the exclusion of relevant reviews or qualitative data. At the eligibility step, the trade-off of authors choosing one SR among many is a loss of potentially important information, which may lead to greater uncertainty about the effects of the intervention, while at the same time removing the issue of discordance.

Overviews of reviews and guidelines with broad clinical, policy or public health questions synthesising results of multiple SRs may choose to weigh all the evidence on the topic and not choose one representative SR. Including all SRs is likely to introduce discordance, and will lead to other challenges when synthesising a large amount of review data (e.g. overlap in primary study data, standardising effect metrics). When including all SRs, resolving these challenges is likely to be resource intensive and cumbersome for the reader. When all SRs are included, authors may compare the effect estimates as we have in Table 3, to determine if there was agreement in the findings (i.e. direction of effect was the same).

### 4.4 Strengths and limitations

Our study has several strengths. First, we used consensus building strategies to develop clear instructions on how to operationalise the Jadad algorithm, and to ensure a consistent approach to assumptions and stepwise interpretation. We also adopted a systematic and transparent approach to address the objectives outlined in our protocol using SR guidance [20]. A comprehensive search strategy, including a search of the grey literature, was employed with no restrictions on language and publication status to minimise publication bias. To minimise error, screening, extractions, and assessments were completed by two independent reviewers, and subsequently compared. Any discrepancies were resolved upon consensus, and when necessary, with the involvement of a third reviewer. Pilot screening and pilot assessments were completed by reviewers and assessed to ensure consistency in understanding of the screening criteria, and definitions of coding and extracted items.

There are some aspects of our methods that should be considered limitations. Our assessments of discordance using the Jadad algorithm were conducted without clinical expertise on some conditions and interventions. We attempted to minimise the impact of this by including both methodologists and clinicians in our research team but given the breadth of topics addressed by these studies, judging the similarity and relevance of clinical, public health, or policy questions and eligibility criteria was at times difficult. It is also possible that a broader search of different databases may have identified more studies using the Jadad algorithm. Our search for overview of reviews (2000–2020) did not focus on identifying Discordant Reviews (i.e. studies aiming to assess discordant results across SRs with similar PICO), therefore we may have missed relevant studies during this period. We recommend that authors trying to identify Discordant Reviews, search for synonyms of discordance in the abstract. This might indicate that the use of the Jadad algorithm might be more prevalent than our study indicates. Findings from this study are not directly generalisable to SRs that include both RCTs and non-RCTs, which would have greater sources of heterogeneity in their study results. Furthermore, our sample is mostly representative of orthopedic conditions and may lack generalisability.

### 4.5 Future research

A tool that has better agreement between decision makers, addresses all pertinent variables that may contribute to discordance, and is easier to implement is needed. Such a tool, whether it be a framework or stepwise algorithm, will need to be applicable to a variety of settings (i.e., SRs of primary studies with and without standard meta-analysis or network meta-analysis). The proposed tool would also need to incorporate recent methodological and statistical advances in evidence synthesis. Validation of such a tool could involve expert opinion obtained from consensus building methods (i.e., Delphi methods) and other methods proposed by Whiting [60] and Moher [61]. Reproducibility will also be important so inter-rater reliability should also be tested. Ideally, such a tool would not only assist the clinician, policy maker, or researcher in choosing the most appropriate SR but guide the user in identifying the most likely sources of discordance.

To address the gap in investigations of discordance using algorithms or methods other than that of Jadad [8], we are currently analysing approximately 70 studies to identify how researchers assessed discordance. We are using a qualitative framework analysis to map out any stepwise approaches used. This study and the current one will inform the development by our team of a newly proposed automated algorithm to assess discordance across SRs with similar clinical, public health, and policy questions, called WISEST (WhIch Systematic rEview iS besT).

Finally, a new tool to assess discordance should take into consideration the conclusiveness, or the stability of the results, of the SRs under question [62–65]. For example, the Cochrane logo shows the summary results from a conclusive SR called “Antenatal corticosteroids for accelerating fetal lung maturation for women at risk of preterm birth” [66]. This review is conclusive, meaning it provides a definitive recommendation for an intervention based on at least one meta-analysis, and has overall consensus among clinicians and/or policy makers worldwide as to its efficacy or effectiveness.

### 4.7 Conclusion

Our results suggest that the Jadad algorithm is not reliably reproducible between decision makers and is inadequate for several reasons. First, there is no comprehensive prescriptive guidance on how to apply the algorithm. Second, quality was not considered when assessing SRs, which represents a major design flaw in the algorithm. Third, this tool fails to incorporate recent advances in biases, methodological and statistical approaches to evidence synthesis.

In the absence of a tool to assess discordance across SRs, we recommend that clinicians, policy makers, patients and researchers address conflicts between review findings by choosing the SR with meta-analysis of RCTs that most closely resemble their question, is the most recent (most recent search date), comprehensive (i.e. number of included RCTs), and is at the lowest risk of bias.

## Electronic supplementary material

Below is the link to the electronic supplementary material.

Supplementary Material 1

## Data Availability

The data is freely available on the Open Science Framework at https://osf.io/bpj2f. The final Jadad algorithm steps, our decision rules and supplementary results are found in the appendices, eight instructional videos are located at https://osf.io/2z7a5/, and our protocol is printed as a preprint on the Research Square server [20].

## Declarations

### Patient involvement

Patients or the public were not involved in the design, or conduct, or reporting, or dissemination plans of our research.

## References

[1] Bornmann L, Mutz R (2015). Growth rates of modern science: A bibliometric analysis based on the number of publications and cited references. J Association Inform Sci Technol.

[2] Bastian H, Glasziou P, Chalmers I (2010). Seventy-five trials and eleven systematic reviews a day: how will we ever keep up?. PLoS Med.

[3] Ioannidis JP (2016). The mass production of redundant, misleading, and conflicted systematic reviews and meta-analyses. Milbank Q.

[4] Taito S (2021). Assessment of the Publication Trends of COVID-19 Systematic Reviews and Randomized Controlled Trials. Annals of Clinical Epidemiology.

[5] Hoffmann F (2021). Nearly 80 systematic reviews were published each day: Observational study on trends in epidemiology and reporting over the years 2000–2019. J Clin Epidemiol.

[6] Bolland MJ, Grey A (2014). A case study of discordant overlapping meta-analyses: vitamin d supplements and fracture. PLoS ONE.

[7] Daei A (2020). Clinical information seeking behavior of physicians: A systematic review. Int J Med Informatics.

[8] Jadad AR, Cook DJ, Browman GP (1997). A guide to interpreting discordant systematic reviews. CMAJ.

[9] Hartling L (2012). A descriptive analysis of overviews of reviews published between 2000 and 2011. PLoS ONE.

[10] Lunny C (2017). Toward a comprehensive evidence map of overview of systematic review methods: paper 1-purpose, eligibility, search and data extraction. Syst Rev.

[11] Lunny C (2018). Toward a comprehensive evidence map of overview of systematic review methods: paper 2-risk of bias assessment; synthesis, presentation and summary of the findings; and assessment of the certainty of the evidence. Syst Rev.

[12] Pieper D (2012). Overviews of reviews often have limited rigor: a systematic review. J Clin Epidemiol.

[13] Bakdach WMM, Hadad R. Effectiveness of different adjunctive interventions in the management of orthodontically induced white spot lesions: A systematic review of systematic reviews and meta-analyses. Dental and Medical Problems; 2020. 10.17219/dmp/118330 33064375

[14] Blom AW (2021). Common elective orthopaedic procedures and their clinical effectiveness: umbrella review of level 1 evidence. BMJ.

[15] Chen P (2019). Intra-articular platelet-rich plasma injection for knee osteoarthritis: a summary of meta-analyses. J Orthop Surg Res.

[16] Li Q (2016). Minimally invasive versus open surgery for acute Achilles tendon rupture: a systematic review of overlapping meta-analyses. J Orthop Surg Res.

[17] Mascarenhas R (2014). Is double-row rotator cuff repair clinically superior to single-row rotator cuff repair: a systematic review of overlapping meta-analyses. Arthroscopy.

[18] Zhao JG, Wang J, Long L (2015). Surgical Versus Conservative Treatments for Displaced Midshaft Clavicular Fractures: A Systematic Review of Overlapping Meta-Analyses. Med (Baltim).

[19] Higgins JP. Cochrane handbook for systematic reviews of interventions version 5.0. 1. The Cochrane Collaboration. 2008. http://www.cochrane-handbook.org.

[20] Lunny C, Kanji TS, Ferri S, Pieper N, Whitelaw D, Thabet S, Tasmin P, Nelson S, Reid H, Zhang E JH., *Identifying and addressing conflicting results across multiple discordant systematic reviews on the same topic: A protocol for a replication study of the Jadad algorithm [Internet]*. 2021: Research Square. Available from: https://www.researchsquare.com/article/rs-931213/v1. 10.1136/bmjopen-2021-054223 PMC9021774 35443948

[21] Lunny C, et al. Bibliometric study of ‘overviews of systematic reviews’ of health interventions: evaluation of prevalence, citation and journal impact factor. Research Synthesis Methods; 2021. 10.1002/jrsm.1530 34628727

[22] Shea BJ (2007). Development of AMSTAR: a measurement tool to assess the methodological quality of systematic reviews. BMC Med Res Methodol.

[23] Shea BJ, et al., *AMSTAR 2: a critical appraisal tool for systematic reviews that include randomised or non-randomised studies of healthcare interventions, or both*. bmj, 2017. 358. 10.1136/bmj.j4008 PMC5833365 28935701

[24] Oxman AD, Guyatt GH (1991). Validation of an index of the quality of review articles. J Clin Epidemiol.

[25] Whiting P, Churchill SJ R. *Introduction to ROBIS, a new tool to assess the risk of bias in a systematic review*. in *23rd Cochrane Colloquium*. 2015. Vienna, Austria: John Wiley & Sons.

[26] Chalmers PN (2015). Do arthroscopic and open stabilization techniques restore equivalent stability to the shoulder in the setting of anterior glenohumeral instability? a systematic review of overlapping meta-analyses. Arthroscopy.

[27] Campbell J, Bellamy N, Gee T (2007). Differences between systematic reviews/meta-analyses of hyaluronic acid/hyaluronan/hylan in osteoarthritis of the knee. Osteoarthr Cartil.

[28] Druyts E (2013). Interpreting discordant indirect and multiple treatment comparison meta-analyses: an evaluation of direct acting antivirals for chronic hepatitis C infection. Clin Epidemiol.

[29] Vavken P, Dorotka R (2009). A systematic review of conflicting meta-analyses in orthopaedic surgery. Clin Orthop Relat Research®.

[30] Poolman RW (2007). Overlapping systematic reviews of anterior cruciate ligament reconstruction comparing hamstring autograft with bone-patellar tendon-bone autograft: why are they different?. J Bone Joint Surg - Am Volume.

[31] Grassi A, et al., *Patellar resurfacing versus patellar retention in primary total knee arthroplasty: a systematic review of overlapping meta-analyses.* Knee Surgery, Sports Traumatology, Arthroscopy, 2018. **26**(11): p. 3206–3218. 10.1007/s00167-018-4831-8 29335747

[32] Erickson BJ (2015). Does operative treatment of first-time patellar dislocations lead to increased patellofemoral stability? A systematic review of overlapping meta-analyses. Arthroscopy: The Journal of Arthroscopic & Related Surgery.

[33] Xiaofeng Chen M (2018). Is unilateral percutaneous kyphoplasty superior to bilateral percutaneous kyphoplasty for osteoporotic vertebral compression fractures? Evidence from a systematic review of discordant meta-analyses. Pain Physician.

[34] Xu B (2017). Is intramedullary fixation of displaced midshaft clavicle fracture superior to plate fixation? Evidence from a systematic review of discordant meta-analyses. Int J Surg.

[35] Song GM (2016). Laparoscopic cholecystectomy for acute cholecystitis: early or delayed?: Evidence from a systematic review of discordant meta-analyses. Med (Baltim).

[36] Tan G, et al., *Unilateral versus bilateral percutaneous balloon kyphoplasty for osteoporotic vertebral compression fractures: A systematic review of overlapping meta-analyses*. Medicine, 2018. 97(33). 10.1097/MD.0000000000011968 PMC6112965 30113502

[37] Xing D (2016). Intra-articular hyaluronic acid in treating knee osteoarthritis: a PRISMA-compliant systematic review of overlapping meta-analysis. Sci Rep.

[38] Mascarenhas R (2015). Does double-bundle anterior cruciate ligament reconstruction improve postoperative knee stability compared with single-bundle techniques? A systematic review of overlapping meta-analyses. Arthroscopy: The Journal of Arthroscopic & Related Surgery.

[39] Houck DA (2017). Early versus delayed motion after rotator cuff repair: a systematic review of overlapping meta-analyses. Am J Sports Med.

[40] Pekala PA (2019). FokI as a genetic factor of intervertebral disc degeneration: a PRISMA-compliant systematic review of overlapping meta-analyses. J Clin Neurosci.

[41] Cui Zhiyong M (2019). Unilateral versus bilateral balloon kyphoplasty for osteoporotic vertebral compression fractures: a systematic review of overlapping meta-analyses. Pain Physician.

[42] Fu Bs (2019). Surgical and Non-Surgical Treatment for 3‐Part and 4‐Part Fractures of the Proximal Humerus: A Systematic Review of Overlapping Meta‐Analyses. Orthop Surg.

[43] Zhao JG (2015). Intramedullary nail versus plate fixation for humeral shaft fractures: a systematic review of overlapping meta-analyses. Med (Baltim).

[44] Guo X-w, et al. Shenyi Capsule (参—胶囊) plus chemotherapy versus chemotherapy for non-small cell lung cancer: a systematic review of overlapping meta-analyses. Chin J Integr Med. 2018;24(3):227–31. 10.1007/s11655-017-2951-5 29043599

[45] Gurevitch J (2018). Meta-analysis and the science of research synthesis. Nature.

[46] *Advances in Evidence Synthesis: special issue Cochrane Database of Systematic Reviews*. Cochrane Database of Systematic Reviews, 2020. 9.

[47] Moher D (2009). Preferred reporting items for systematic reviews and meta-analyses: the PRISMA statement. PLoS Med.

[48] Page MJ (2021). Updating guidance for reporting systematic reviews: development of the PRISMA 2020 statement. J Clin Epidemiol.

[49] Brożek J (2009). Grading quality of evidence and strength of recommendations in clinical practice guidelines: part 1 of 3. An overview of the GRADE approach and grading quality of evidence about interventions. Allergy.

[50] BMJ (2021). Multiple systematic reviews on the same question.

[51] Robinson KA (2008). AHRQ Methods for Effective Health Care Integrating Bodies of Evidence: Existing Systematic Reviews and Primary Studies. Methods Guide for Effectiveness and Comparative Effectiveness Reviews.

[52] Masic I, Miokovic M, Muhamedagic B. *Evidence based medicine - new approaches and challenges.* Acta informatica medica: AIM : journal of the Society for Medical Informatics of Bosnia & Herzegovina : casopis Drustva za medicinsku informatiku BiH, 2008. 16(4): p. 219–225. 10.5455/aim.2008.16.219-225 PMC3789163 24109156

[53] Mašić I. Porodicna/obiteljska medicina: principi i praksa. na; 2007.

[54] Arrich J (2005). Intra-articular hyaluronic acid for the treatment of osteoarthritis of the knee: systematic review and meta-analysis. CMAJ.

[55] Lo GH (2003). Intra-articular hyaluronic acid in treatment of knee osteoarthritis: a meta-analysis. JAMA.

[56] Bellamy N, et al., *Viscosupplementation for the treatment of osteoarthritis of the knee*. Cochrane database of systematic reviews, 2006(2). 10.1002/14651858.CD005321.pub2 PMC8884110 16625635

[57] Wang C-T (2004). Therapeutic effects of hyaluronic acid on osteoarthritis of the knee: a meta-analysis of randomized controlled trials. JBJS.

[58] Raynauld J (2005). Effectiveness and safety of repeat courses of hylan GF 20 in patients with knee osteoarthritis. Osteoarthr Cartil.

[59] Raynauld J-P (2002). A prospective, randomized, pragmatic, health outcomes trial evaluating the incorporation of hylan GF 20 into the treatment paradigm for patients with knee osteoarthritis (Part 1 of 2): clinical results. Osteoarthr Cartil.

[60] Whiting P (2017). A proposed framework for developing quality assessment tools. Syst Rev.

[61] Moher D (2010). Guidance for developers of health research reporting guidelines. PLoS Med.

[62] Babić A (2020). How to decide whether a systematic review is stable and not in need of updating: Analysis of Cochrane reviews. Res Synthesis Methods.

[63] Cohen S (2014). Conclusiveness of the Cochrane reviews in nutrition: a systematic analysis. Eur J Clin Nutr.

[64] Dosenovic S (2020). Reasons and factors associated with inconclusiveness of systematic reviews about interventions for neuropathic pain. J Comp Eff Res.

[65] Mimouni M, Mimouni F, Segev F (2015). Conclusiveness of the Cochrane eye and vision group reviews. BMC Res Notes.

[66] Roberts D, et al., *Antenatal corticosteroids for accelerating fetal lung maturation for women at risk of preterm birth*. Cochrane database of systematic reviews, 2017(3). 10.1002/14651858.CD004454.pub3 PMC6464568 28321847

