# Supplementary material for: How can clinicians choose between conflicting and discordant systematic reviews? A replication study of the Jadad algorithm
Source: BMC Med Res Methodol. 2022 Oct 26;22:276. doi: 10.1186/s12874-022-01750-2 (PMC9597955; doi:10.1186/s12874-022-01750-2)
Supplement: Supplementary file 1 — Supplementary Material 1 [file 12874_2022_1750_MOESM1_ESM.pdf]

Appendices

Appendix A: Jadad algorithm interpretation and instructions
Appendix B: Data extraction methods
Appendix C. Deviations to our protocol
Appendix D: Discordant reviews that did not choose one systematic review as the best evidence
Appendix E: Case study of the clinical impact of choosing one systematic review using the Jadad algorithm
Appendix F: How easy were the Jadad steps in interpreting and operationalising? (Ease of use)
Appendix G: Time and ease of use in completing Steps H and I of the Jadad assessments
Appendix H: ROBIS Results and Table

Appendix A
Jadad algorithm interpretation and instructions

Extraction of the primary intervention and outcome

1.1 Identification of the primary outcome from the discordant review

As a first step in assessing discordance, we will identify the primary outcome from each discordant review. The primary outcome will be extracted when it is explicitly defined in the title, abstract, objectives, introduction or methods section. If the primary outcome is not found by this means, we will extract it as the outcome that is the first outcome mentioned in the manuscript.

Save the citation of the discordant review in Zotero Web library https://www.zotero.org/

- Create a new folder in Zotero with the first author year as the tile of the folder
- Save the discordant review citation to this folder

Do I extract information from the discordant review or the included systematic reviews (SRs)?

- Extract information FIRST from the discordant review
- If the data you need is not found in the discordant review, then search in the included SRs with meta-analyses.
- If the information in the SR and discordant review is different/discrepant, then note this in the notes section.

1.2 Identification of the primary intervention/comparison from the discordant review

If multiple interventions are present, we will choose the first intervention highlighted in the title or abstract of the discordant review that aligns with the primary outcome [1].

1.3 Identification of included SRs with meta-analysis of RCTs addressing the primary intervention/outcome of the discordant review

We will next identify how many SRs with meta-analysis of RCTs were included in the discordant review that address the primary outcome. It is this sample of SR with meta-analysis of RCTs that will be the focus of our Jadad assessments. To identify a systematic review with meta-analysis of RCTs, you should see if this information is in the discordant review. It is not enough for it to say "systematic review with meta-analysis" because it could be a meta-analysis of only observational studies, or mixing RCTs with observational studies.

Next go to the individual systematic review with meta-analysis to the methods selection of studies, and the results sections. There they will state which study types they included in the methods section, and what they found in the results. Identify if RCTs were included, and then check the forest plot for the primary intervention/outcome to see if they did a meta-analysis grouping only RCTs (which is good, then it can be included), or if they mixed RCTs with observational studies (e.g. cohort, case control, quasi-experimental, cross-sectional) (which is bad, then exclude).

Meta-analysis with RCTs grouped and providing a pooled effect estimate are included

Example of a good meta-analysis where the authors grouped the RCTs and presented:

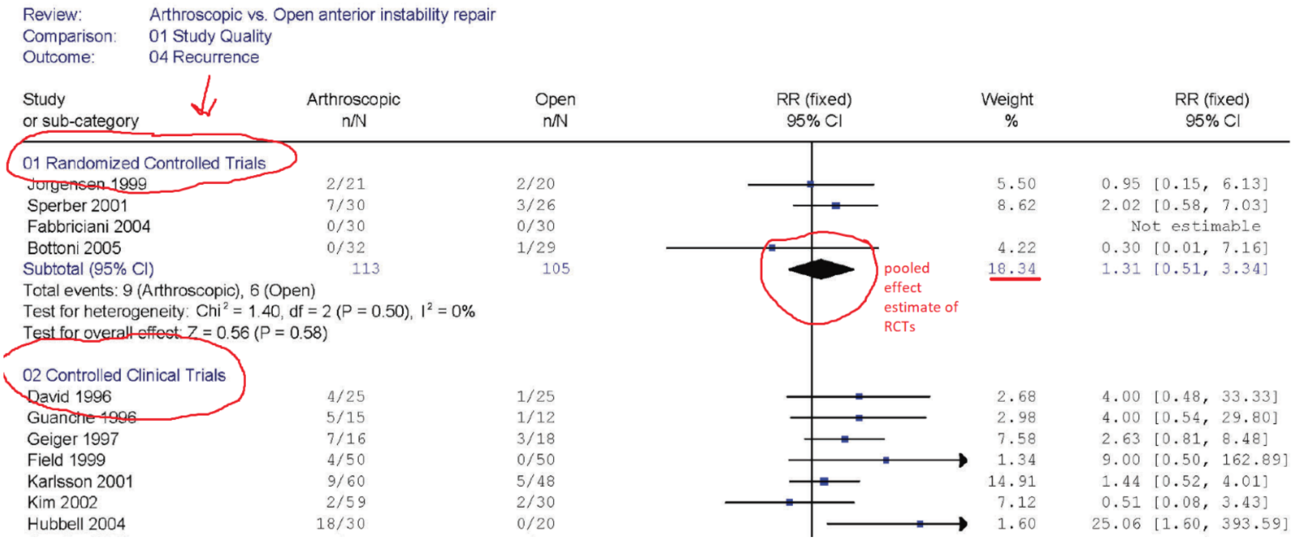

Meta-analysis mixing study designs are excluded

Example of meta-analysis mixing RCTs with different study designs in the same analysis. This is wrong. You keep all study designs separate in a meta-analysis.

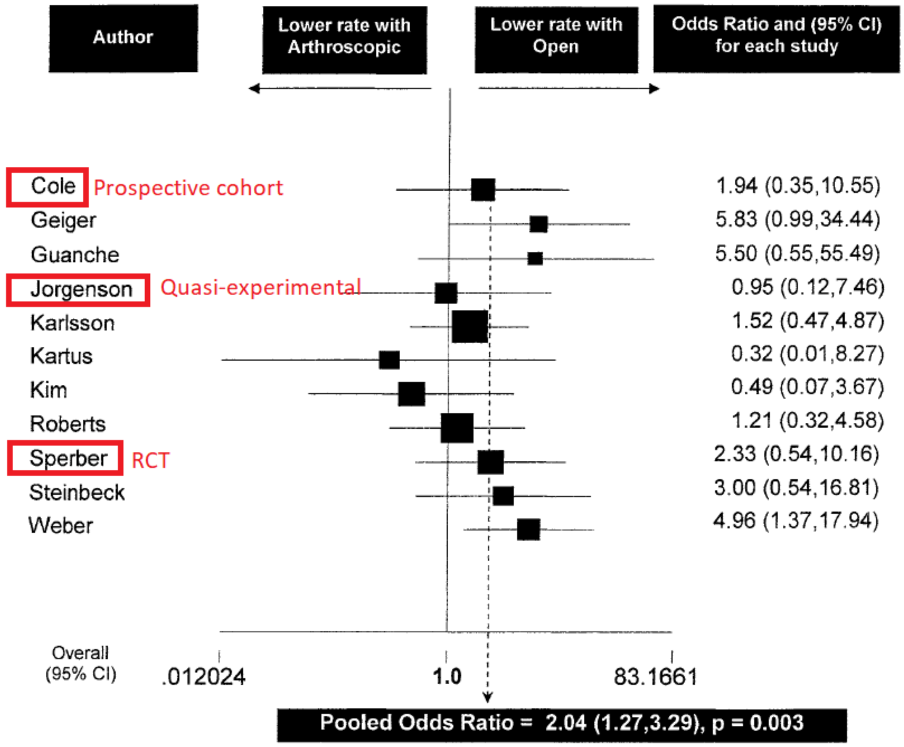

FIGURE 1. Mantel-Haenzel Pooled odds ratio for recurrent instability. The solid vertical center line represents an odds ratio of 1.0.

Not a meta-analysis

In addition, many older studies may say they are systematic reviews with meta-analysis in the title or abstract, but they do not conduct a statistically correct meta-analysis using random or fixed effects model that accounts for within study heterogeneity and proper weighting of the studies. For example, the Chalmers discordant review includes two “meta-analyses” that are not meta-analyses – they use linear regression which is not the

correct methodology (see Freedman 2004). This is true for a majority of "meta-analyses" published prior to 2011.

Save the citation of the SRs in Zotero Web library

- Save the SR citations to the discordant review folder

Data extraction form

- Record the primary outcome, primary intervention/comparison, #SRs, SR citations, and # RCTs in each SR that addresses the primary outcome in the excel file

1.4 Process to identify primary intervention and outcome

One author will extract the primary intervention and outcome, which will be checked by a second author, and disagreements will be discussed until consensus is reached.

1.5 Blinding of results in the included SRs with meta-analysis of RCTs

We will blind the following components containing study results of the Jadad assessment and conclusions: abstract, highlights, results of the Jadad assessment, and discussion/conclusions section. Blinding will be achieved via deletion using the paywalled Adobe Acrobat Pro or the freeware PDFCandy (https://pdfcandy.com). One author will blind the discordant review results and will not be involved in the Jadad assessment pertaining to those results.

Save the blinded discordant review to the Zotero folder with the discordant review author first name and year.

1.6 Piloting Jadad assessment

A pilot practice exercise will be conducted by all assessors prior to the Jadad assessments, to ensure consistent assessments across reviewers. One to three Jadad assessments will be piloted by each reviewer and compared to a second to identify discrepancies and resolve through discussion.

1.7 Jadad assessments of discordance

Two researchers will independently assess each set of SRs in the included discordant review using the Jadad algorithm, starting with Step C (**Supplementary Figure 1 below**).

Step A is to examine the multiple reviews matching the discordant review question using a PICO framework. If the research questions are not identical, then step B indicates choosing the review closest to the decision makers' research question and no further assessment is necessary. If multiple reviews are found with the same PICO, then Step C should be investigated. As we are using discordant reviews examining discordance as our sample, we will start at Step C in the Jadad decision tree.

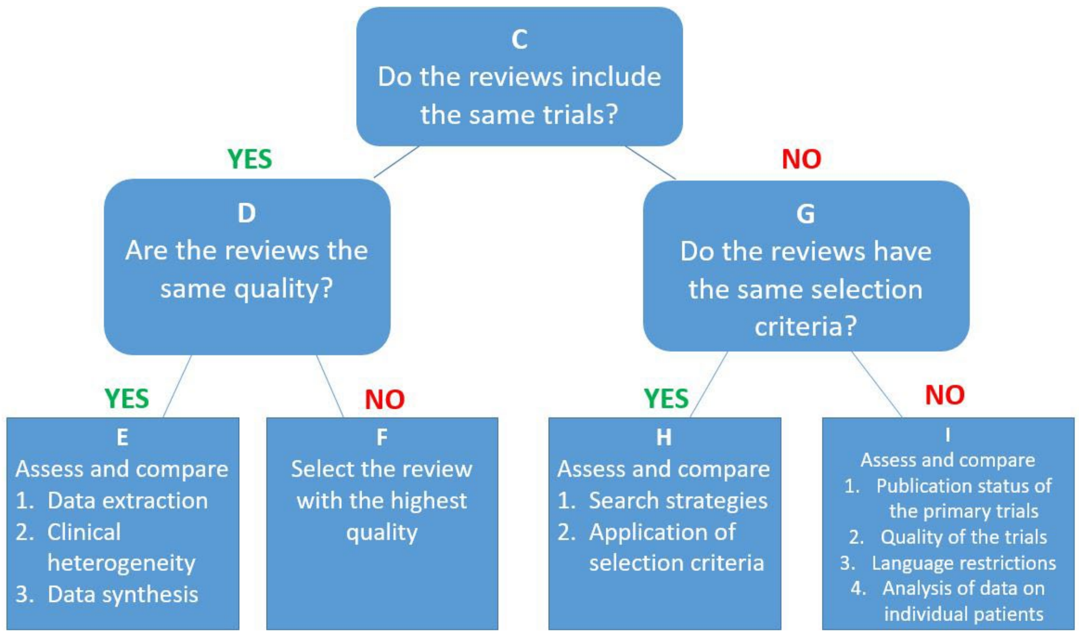

Supplementary Figure 1. Jadad (1997) decision tree.

Legend: Step A is to examine the multiple reviews matching the decision makers' question using a PICO framework. If the research questions are not identical, then step B indicates choosing the review closest to the decision makers' research question and no further assessment is necessary. If multiple reviews are found with the same PICO, then step C should be investigated.

Here we detail our interpretation of the Jadad algorithm for each step in assessing the discordance in a group of SRs with similar PICO elements. If a discordant review or the included SR does not report a method, we will indicate it as "not reported", and it will not be chosen for that step.

Steps D and G follow from Step C. Steps E, F, H. and I are completed depending on the decisions at Steps D and G respectively.

- Record all data you extract in the excel file.
- Instructions for filling out the excel form: For the Data rows, fill in the drop -down boxes. Notes column contains quotes from the study to justify the rationale for each drop -down box decision you make. Please provide a rationale if the decision is not clear cut.
- Please use the data from the Bakdach discordant review to do your Jadad assessment FIRST. You might have to search for data in the included SRs, but if you do, please note it in the notes sections, as this will impact on the "ease of use".

STEP C

2.1 Step C - Do the reviews include the same trials pertaining to the primary intervention and outcome?

We will determine if the RCTs are all the same (identical) across reviews by either finding this information in the discordant review, or extracting all RCTs from the included reviews using an excel matrix to list the reviews at the top, and trials in the left rows. If you have to find this information in the included SRs, then look for the forest plot of the primary outcome and the primary intervention.

The RCTs will be mapped to the reviews in order of publication date (earliest trials at the top). Using this matrix, we will determine if the reviews include the same or different trials.

Instructions:

- Check the discordant review manuscript and appendices (i.e. supplementary material), to see if the discordant review authors mapped the trials to each SR in a matrix. Must be RCTs pertaining to our intervention and outcome of interest!
- Look first for the meta-analysis forest plot for the primary intervention and outcome in the SR manuscript pertaining to the intervention and outcome of interest. From the forest plot, note the RCTs by first author's last name and date of publication.
- Epistemonikos sometimes maps the SRs included in a discordant review, and the trials included in each SR. You can check Epistemonikos.org to see of the discordant review is included in the database and contains a matrix. Put the title in the search engine.
- If the discordant review authors list the RCTs for each SR but do not provide a matrix, then make a judgment based on the list of RCTs by SR pertaining to our intervention and outcome of interest.
- If no, you will then create a matrix using Excel with trials on the top (columns) and systematic reviews in the rows. The coloured boxes represent the trials included in each SR. Example:

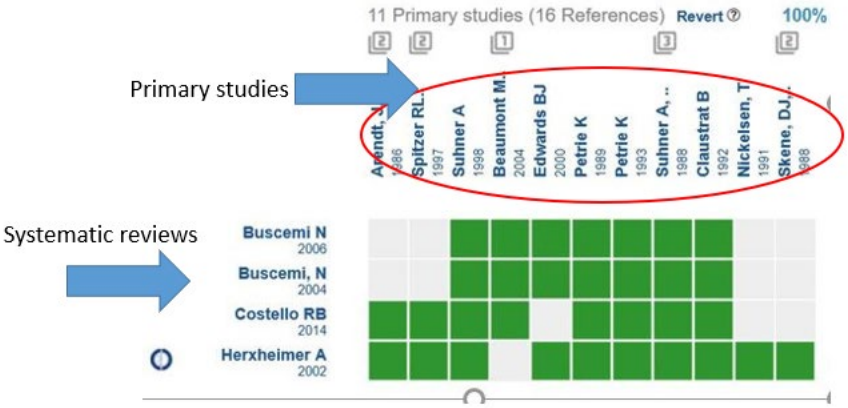

- Record your results in the excel file on whether the reviews contain the same trials for our intervention and outcome of interest
- It may be the case that 2 SRs contain the same trials, but the third SR does not. If this is the case, then the SRs do not contain the same trials.

STEP G

3.1 Step G - Do the reviews have the same eligibility criteria?

If the reviews do not include the same trials, then decision-makers are directed to turn to Step G – assess whether the reviews have the same eligibility criteria. The discordant review may contain text in a methods section, or characteristics of included reviews table where the PICO eligibility criteria can be extracted and assessed. If this is not the case, then the PICO eligibility criteria will be extracted from the included reviews by two authors independently and then compared to resolve any discrepancies.

Instructions:

- **Fill in the excel form by copying and pasting quotes from the discordant review or reviews, then make a decision about the overall assessment of step G**

FOR THE PILOT JADAD ASSESSMENTS, FILL IN ALL THE STEPS/ITEMS IN THE EXCEL FILE. RECORD THE STEPS YOU WOULD TAKE TO CHOOSE THE REVIEW IN THE TAB CALLED "JADAD ASSESSMENT" BY COLOUR FILL THE BOXES WITH THE STEP YOU WOULD CHOOSE.

EXAMPLE:

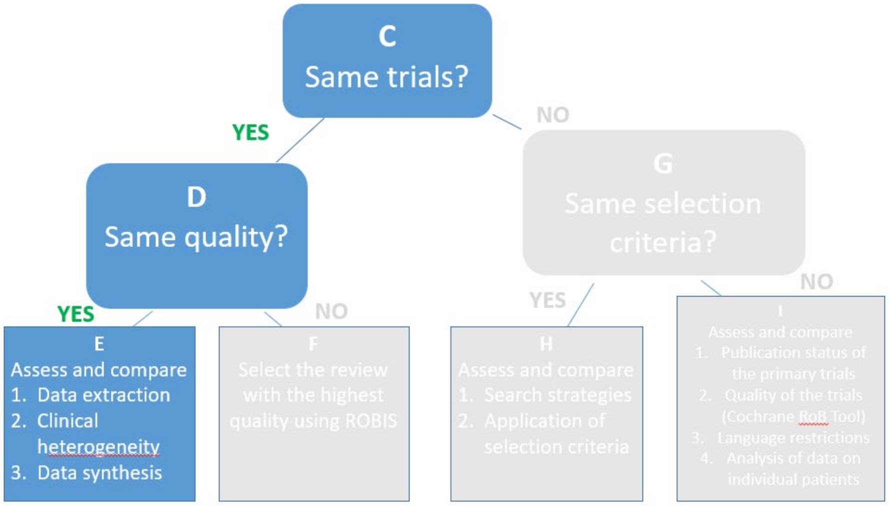

The steps chosen in this Jadad assessment are highlighted in blue, and the other steps greyed out. We therefore deduce that Step D then Step E were used to choose a review.

While doing your Jadad assessment, make notes about how discordant review authors used Jadad and if they adhered strictly to our Jadad instructions, and if no, how they changed it, how was it different than our instructions. This will be recorded in the excel file.

MAKE SURE TO TIME YOURSELF

- Look for answers in the discordant review first, and only if the answer can't be found, then look in the reviews.
- Make sure you add a quote from the discordant review or review (stating which it is from), and your rationale if its not clear

STEP D

4.1 Step D - Are the reviews of the same quality?

If the reviews contain the same trials, then the decision-maker moves to Step D – assess whether the reviews are of the same methodological quality. We will either (a) extract the quality assessments from the discordant review if the discordant review used AMSTAR [2], AMSTAR 2 [3], or ROBIS [4]. If the discordant review authors used any other tool or method to assess the risk of bias/quality of the reviews, or did not assess the risk of bias/quality assessment at all, we will do our own assessment using the ROBIS tool [4]. We anticipate that most discordant reviews will have used AMSTAR or AMSTAR-2, therefore please make a judgment about the **quality** of the SRs.

Instructions:

- **Check the discordant review manuscript and appendices (i.e. supplementary material), to see if the discordant review authors assessed the quality of the included SRs.**
- Report the judgment of quality in the excel file.
- If no, conduct an assessment of risk of bias of each SR using ROBIS and report your overall judgment (e.g. Low risk, High risk, Unclear risk)

If the discordant review does not provide an overall quality for each SR, put "not reported", then use this decision rule for AMSTAR and AMSTAR-2 found below.

AMSTAR. To be high quality, the review must have answered yes to the following questions. To choose the study with the highest quality, pick the review with the most YES answers to the Yes questions below:

1. A priori design	YES
2. Duplicate study selection	YES
2. Duplicate data extraction	YES
3. Comprehensive literature search	YES
4. Status of publication used as an inclusion criterion	YES
5. List of included studies	YES
5. List of excluded studies	NO
6.Characteristics of included studies reported	NO
7. Quality of studies assessed	YES
8. Quality of studies used appropriately in formulating conclusions	YES
9. Appropriate methods used to combine results	YES
10. Publication bias assessed	NO
11. Conflict of interests noted for review	NO
11. Conflict of interests noted for included studies	NO

AMSTAR-2. To be high quality, the review must have answered yes to the following questions

1. Did the research questions and inclusion criteria for the review include the components of PICO?	YES
2. Did the report of the review contain an explicit statement that the review methods were established prior to the conduct of the review (ie was there a protocol written), and did the report justify any significant deviations from the protocol?	YES
3. Did the review authors explain their selection of the study designs for inclusion in the review?	YES
4. Did the review authors use a comprehensive literature search strategy?	YES
5. Did the review authors perform study selection in duplicate?	YES
6. Did the review authors perform data extraction in duplicate?	YES
7. Did the review authors provide a list of the excluded studies and justify the exclusions?	No
8. Did the review authors describe the included studies in adequate detail?	YES
9. Did the review authors use a satisfactory technique for assessing the risk of bias (RoB) in individual studies that were included in the review?	YES
10. Did the review authors report on the sources of funding for the studies included in the review?	No
11. If meta-analysis was performed, did the review authors use appropriate methods for the statistical combination of the results?	YES
12. If meta-analysis was performed, did the review authors assess the potential impact of RoB in individual studies on the results of the meta-analysis or other evidence synthesis?	YES
13. Did the review authors account for RoB in individual studies when interpreting/discussing the results of the review?	YES
14. Did the review authors provide a satisfactory explanation for, and discussion of, any heterogeneity observed in the results of the review?	YES
15. If they performed quantitative synthesis, did the review authors carry out an adequate investigation of publication bias (small-study bias) and discuss its likely impact on the results of the review?	No
16. Did the review authors report any potential sources of conflict of interest, including any funding they received for conducting the review?	No

STEP F

5.1 Step F - Select the review with the highest quality

From the risk of bias/quality assessment conducted through Step D, we will choose the review with the highest quality assessment rating. We anticipate that most discordant reviews will have used AMSTAR or AMSTAR-2, therefore please make a judgment about the **quality** of the SRs.

For our Jadad assessment, we will choose a binary rating of either high quality or low quality. When using the assessments of quality of reviews from the included discordant reviews, we will choose the rating of the authors. If uncertainty exists, we will re-assess the included reviews using ROBIS and convert to quality (e.g. low risk = high quality).

Instructions:

- **Fill in the excel form, then make a decision about the overall assessment of step F**

STEP E

6.1 Step E – Assess and compare data extraction, clinical heterogeneity, and data synthesis

If the reviews are the same quality, then the next step is Step E, to assess and compare data extraction, clinical heterogeneity, and data synthesis across the reviews.

Instructions:

- **Fill in the excel form, then make a decision about the overall assessment of step E**

Step E1 - Assess and compare the data extraction methods across reviews

For this step, Jadad states "If reviews differ [in outcomes reported], the decision maker should identify the review that takes into account the outcome measures most relevant to the problem that he or she is solving". We interpret this step as selecting the review that (A) matches the discordant review's primary outcome. As we already assessed that the discordant review primary outcome matches the SRs primary outcomes, this item is always answered YES. This cell will be automatically populated.

Jadad then writes that reviews that conduct independent extraction by 2 reviewers are the highest quality. We therefore decided that reviews that (B) used an independent data extraction process using 2 review authors. If a ROBIS assessment is done, then the latter point will be mapped to ROBIS 3.1. Were efforts made to minimise error in data collection? AMSTAR item 3, and AMSTAR-2 item 2b.

Decision rules.
#1. Reviews that meet criteria A and B are highest in our hierarchy
#2. Reviews that meet criteria A are second highest in our hierarchy
#3. Reviews that meet criteria B are third highest in our hierarchy

Step E2 – Assess and compare clinical heterogeneity of the included RCTs across reviews

Clinical heterogeneity is assessed at the review level by examining the research question pertaining to the primary outcome and the eligibility criteria PICO elements of each included RCT to see if they are sufficiently similar. If the PICO across RCTs are similar, then clinical heterogeneity is minimal, and reviews can progress with pooling study results in a meta-analysis. If a ROBIS assessment is done, this question is mapped to ROBIS 4.3 Was the synthesis appropriate given the nature and similarity in the research questions, study designs and outcomes across included systematic reviews? It also maps to AMSTAR-2 item 14, even though AMSTAR-2 does not state whether they mean clinical or statistical heterogeneity. As high statistical heterogeneity is always a result of clinical heterogeneity/variability.

If a review states that (A) they assessed the clinical (PICO) heterogeneity across RCTs (in the methods or results sections), then this will be the review that is chosen at this step. Reviews can also include a characteristic of studies table with PICO elements, or may have described variability in the PICO across RCTs in the results or discussion sections. Example of a review reporting a clinical heterogeneity assessment: "If we found studies with similar study populations, treatment interventions, and outcome assessments, we conducted quantitative analyses." These items are just about whether they DID this, not about whether they did it correctly.

If authors commented on clinical heterogeneity, then rule (B) the clinical heterogeneity assessment was determined to be minimal or low or if it was high, was it addressed in the synthesis? (look for statements like "variability among studies was low", PICO were similar", homogeneity, clinical heterogeneity) HINT: AMSTAR-2 item 14 addresses this, and ROBIS item 4.4. Example: "For both hip and nonvertebral fracture prevention by vitamin D, our pooled results indicated variation between studies that was resolved when low-and high dose vitamin D (cholecalciferol) trials were pooled separately."

Decision rule:
#1. Reviews that meet criteria A and B are highest in the hierarchy
#2. Reviews that meet criteria A are second highest in our hierarchy

Step E3 – Assess and compare data analysis methods across reviews
Jadad et al. are purposefully vague when describing how to judge whether a meta-analysis was appropriately conducted. For this step, we interpret it as reviews reported conducting an: (A) weighted technique to combine study results (i.e. used a fixed or random random-effects model), and (B) whether authors conducted an investigation of statistical heterogeneity (i.e. by reporting I2, tau2 or chi2). These items are just about whether they DID this, not about whether they did it correctly. For criteria B on whether statistical heterogeneity was conducted, if its not stated in the methods, you can also look in the individual SR's forest plot to see if its reported there.

Decision rules for if the presence or absence of heterogeneity is present in the meta-analysis:
#1. Reviews that meet criteria A and B are highest in our hierarchy
#3. Reviews that meet criteria A only are second highest in our hierarchy
#6. Reviews that meet criteria B only are third highest in our hierarchy (this decision can be ignored if heterogeneity is not observed)

Select the review that has the highest ranking in each step. If the reviews only meet partial steps, then choose the review meeting the most criteria. This will be the review that "meets the criteria" for each step. If no criteria are met, then put "criteria not met".

Decision rules for Step E:

#1. Reviews that meet Step E1, E2, and E3 are highest in our hierarchy
#2. Reviews that meet Step E1 and E2 second highest in our hierarchy
#3. Reviews that meet Step E1 third highest in our hierarchy
#4. Reviews that meet Step E2 and E3 fourth highest in our hierarchy
#5. Reviews that meet Step E2 fifth highest in our hierarchy
#6. Reviews that meet Step E3 sixth highest in our hierarchy
Note: Reporting only Steps E1, E2 or E3 is not considered a systematic approach to evidence synthesis.

STEP H

7.1 Step H - Assess and compare the search strategies and the application of the eligibility criteria across reviews

If the reviews contain the same eligibility criteria, then Step H is to assess and compare the search strategies and the application of the eligibility criteria across reviews.

- **Fill in the excel form by copying and pasting quotes from the reviews, then make a decision about the overall assessment of step H**

Step H1 - Assess and compare the search strategies across reviews
In this step, Jadad et al.'s recommendations are vague, although they make reference to comprehensive search strategies as being less biased. We interpret this step as authors explicitly describing their search strategy such that it can be replicated. To meet this interpretation, our criteria are that reviews: (A) search 2 or more databases, (B) search the grey literature (auto-populated cell in the excel form from E1-B); and (C) include a full search algorithm attached as an appendix to the review.

Decision rules:
#1. Reviews that meet criteria A, B and C are highest in our hierarchy
#2. Reviews that meet criteria A and B are second highest in our hierarchy
#3. Reviews that meet criteria A and C are third highest in our hierarchy
#4. Reviews that meet criteria B and C are fourth highest in our hierarchy (unlikely scenario)
#5. Reviews that meet criteria A only are fifth highest in our hierarchy

<u>SCENARIOS for Step H1</u>
• 3 reviews are identified for our Jadad assessment
Criteria to choose a systematic review at Step H1: (A) 2 or more databases – (B) searched grey literature --(C) full search in appendix

Scenario 1
Review 1: A and B but not C
Review 2 does A and B but not C
Review 3 does A and C, but not C
Conclusion: No review meets ALL of our criteria; which do we choose? Based on our decision rules, we choose <u>BOTH</u> Review 1 and 2

Scenario 2
Review 1: A, but not B or C
Review 2: A and B, but not C
Review 3: Does not do A, B, or C (does not report the search methods)
Conclusion: No review meets ALL of our criteria; which do we choose? Based on our decision rules, we choose Review 2

Step H2 - Assess and compare the application of the eligibility criteria across reviews
In this sub-step, Jadad indicates that decision-makers should choose the review with the most explicit and reproducible inclusion criteria, which is ambiguous. Jadad states "Reviews with the same selection criteria may include different trials because of differences in the application of the criteria, which are due to random or systematic error. Decision makers should regard as more rigorous those reviews with explicit, reproducible inclusion criteria. Such criteria are likely to reduce bias in the selection of studies" [5]. We did not know if this meant clearly reproducible PICO eligibility criteria, as this would be a repeat to Step G, whether the eligibility criteria were applied consistently by reviews (i.e. compare eligibility criteria to include RCTs' PICO to see if they indeed met the eligibility criteria), or if this meant (A)

independently screening of title, abstracts and full text against the eligibility criteria by two reviewers. We selected the latter criteria when choosing from the included reviews in a discordant review.

Decision rules:
#1. Reviews that meet criteria A is the highest in our hierarchy

Select the review that has the highest ranking in each step. If the reviews only meet partial steps, then choose the review meeting the most criteria. This will be the review that meets the criteria for each step. If no criteria are met, then put "criteria not met".

Decision rules for Step H:

#1. Reviews that meet Step H1 and H2 highest in our hierarchy
#2. Reviews that meet Step H1 second highest in our hierarchy
#3. Reviews that meet Step H2 third highest in our hierarchy

STEP I

8.1 Step I – Assess and compare the publication status, quality, language restrictions of the included RCTs, and analysis of data on individual patients

If the reviews do not have the same eligibility criteria, then the next step is Step I, to assess and compare the publication status, quality, language restrictions of the included RCTs, and analysis of data on individual patients across the reviews. This step maps to ROBIS item 1.5, namely, were any restrictions in eligibility criteria based on appropriate information sources (e.g. publication status or format, language, availability of data) [4]?

- **Fill in the excel form by copying and pasting quotes from the reviews, then make a decision about the overall assessment of step I**

Step I1 – Assess and compare the publication status of the included RCTs across reviews

In the absence of clear guidance, we interpret this step as "choose the review that searches for and includes both published and unpublished data (grey literature)." Published studies are defined as any study or data published in a peer-reviewed medical journal. You can tell if they have included and searched for published studies by the databases they search such as Pubmed/Medline, Embase, PsycInfo, Cinhal, and Cochrane registry of trials (this is a database of published trials). Any Cochrane registry is a database of published trials. Basically we want to know if the review searched the grey literature (unpublished studies).

Unpublished data is defined as any information that is difficult to locate and obtained from non-peer-reviewed sources such as websites (e.g. World Health Organisation website, CADTH), clinical trial registries (e.g. clinicaltrials.gov), searching for unpublished protocols in PROSPERO, thesis and dissertation databases, abstracts of conferences, handsearching conferences, and other unpublished data registries (e.g. LILACS). Our interpretation is that reviews that indicate in the eligibility section of the methods, that the authors included: (A) studies published in peer-reviewed medical journals, and included (B) reports/documents/content that are not published in medical journals.

Decision rules:
#1. Reviews that meet criteria A and B are highest in our hierarchy
#2. Reviews that meet criteria A are second highest in our hierarchy
#3. Reviews that meet criteria B are third highest in our hierarchy
Note: Reporting only A or B is not considered a systematic search.

Step I2 – Assess and compare the methods used to assess the quality of the included RCTs across reviews

In this step, the Jadad paper recommends assessing the appropriateness of the *methods used* to assess the quality of the included RCTs across reviews. This item maps to ROBIS item 3.4, was the risk of bias/quality of RCTs formally assessed using appropriate criteria? Here we interpret this item as meaning the review authors used the Cochrane risk of bias tool (version 1 or 2). All other RCT quality assessment tools are inappropriate because they are out of date and omit important biases (e.g. Agency for Healthcare Research and Quality (AHRQ) 2012 [6]). However, the Cochrane risk of bias tool was only published in October 2011. Therefore, we applied a decision rule: for reviews dated 2012 (giving one year for awareness of the tool to reach researchers) and later, the Cochrane risk of bias tool is considered the gold standard. For reviews dated 2011 or earlier, we considered the Jadad 1998 scale [7] and Schulz 1995[8] to be the most common scales used between 1995 and 2011. Other tools will be considered on a case-by-case basis.

- 1995-2011 – Jadad scale for RCTs, or other scales
- 2012 – 2021 - Cochrane RoB tool v1 or v2

HINT: Look at the reference used for the assessment of quality and put in the notes

As a decision hierarchy, to meet the minimum criteria for this step, a review will have (A) assessed the risk of bias of RCTs using any tool or approaches, and (B) used the Cochrane risk of bias tool v1 or 2 (if dated 2011 or later). If several reviews are included that meet these two criteria, the review that (C) integrates the risk of bias assessments into the results or discussion section (i.e. discusses risk of bias in terms of high and low risk of bias studies, reports a subgroup or sensitivity analysis) will be chosen.

Decision rules:
#1. Reviews that meet criteria A, B and C are highest in our hierarchy
#2. Reviews that meet criteria B and C are second highest in our hierarchy
#3. Reviews that meet criteria A and B are third highest in our hierarchy
#4. Reviews that meet criteria A and C are fourth highest in our hierarchy (unlikely scenario)
#5. Reviews that meet criteria A only are fifth highest in our hierarchy

SCENARIOS for Step I2• 3 reviews are identified for our Jadad assessment

Scenario 1
Review 1: A and B but not C (third)
Review 2: A and B but not C (third)
Review 3: A and C, but not B (fourth)
Conclusion: No review meets ALL of our criteria; which do we choose? Based on our decision rules, we choose BOTH Review 1 and 2

Scenario 2
Review 1: A, but not B or C (fifth)
Review 2: A and B, but not C (third)
Review 3: Does not do A, B, nor C (does not report the search methods)
Conclusion: No review meets ALL of our criteria; which do we choose? Based on our decision rules, we choose Review 2

Step 13 - Assess and compare any language restrictions across reviews

In this step, Jadad indicates that reviews with (A) no language restrictions in eligibility criteria should be prioritised and chosen over those that only include English language RCTs. This step maps to ROBIS item

1.5, namely, were any restrictions in eligibility criteria based on sources of info appropriate (e.g. publication status or format, language, availability of data)?

Decision rule:
#1. Reviews that meet criteria A are highest in our hierarchy

Step I4 – Choose the analysis of data on individual patients
If (A) an individual patient data (IPD) meta-analysis was identified in the discordant review, Jadad et al. recommend this review be chosen over reviews with pairwise meta-analysis. If the review is an IPD meta-analysis it will say so in the title most likely, and then if not, read the abstract. If it does not state it in either, then assume it is NOT an IPD meta-analysis. Read here for more info about this type of study design: **https://methods.cochrane.org/ipdma/about-ipd-meta-analyses**

Decision rule:
#1. Reviews that meet criteria A are highest in our hierarchy

Select the review that has the highest ranking in each step. If the reviews only meet partial steps, then choose the review meeting the most criteria. This will be the review that meets the criteria for each step. If no criteria are met, then put "criteria not met".

Decision rules for Step I:
#1. If there is an IDP meta-analysis (Step I4) then this review is the highest in our hierarchy
#2. Reviews that meet Step I1, I2, and I3 are second highest in our hierarchy
#3. Reviews that meet Step I1 and I2 third highest in our hierarchy
#4. Reviews that meet Step I2 and I3 fourth highest in our hierarchy
#5. Reviews that meet Step I1 and I3 fifth highest in our hierarchy
#6. Reviews that meet Step I1 sixth highest in our hierarchy
#7. Reviews that meet Step I2 seventh highest in our hierarchy
#8. Reviews that meet Step I3 eighth highest in our hierarchy

9.1 Study outcomes

How much time did it take you to do this Jadad assessment? (in minutes)

- Stope timing yourself here and note how long the assessment took in minutes

Evaluation of whether the discordant review authors Examined and recorded reasons for discordance

- **In the excel form, record if the discordant review authors used the same instructions as us to do their Jadad assessment, i.e. did they interpret each step the same as we did in our study? If no, describe the differences between our interpretation of the Jadad steps, and the discordant review authors interpretation (at the stage of the step that is different).**

Ease of use rating
Was this Jadad assessment easy, moderate or hard to do? (Ease of use rating), and add your rationale in the notes.

Easy	The step can be accomplished easily by the reviewer, due to low cognitive load or because it's a recognised method or approach
Moderate	The step requires a notable degree of cognitive load by the reviewer but can generally be accomplished with some effort
Hard	The step is difficult for the reviewer, due to significant cognitive load or confusion; some reviewers would likely fail or abandon the task at this point

Based on your Jadad assessment, you now need to decide how easy, moderate or hard it was to fill out and assess.

- Write your rationale for your choice in the notes section.
- Prompt questions: which step did you find challenging and why?
- How was this set of SRs hard to assess?

Which review(s) was chosen by the discordant review authors?

- Find the discordant review online and determine which review(s) was/were chosen by the authors based on their Jadad assessment

What review do YOU think should have been chosen as the best review and why?

- Based on your assessment do you have an opinion about which review should have been chosen? If yes, which one and why?

Appendix B
Data extraction methods
Outcome extraction

1. Study characteristics (lead author's name, publication year).
2. Research question (objectives, health condition of treatment, PICO eligibility criteria: participant, intervention/comparison, and primary outcome).
3. Methods (how the discordant review authors assessed discordance among the reviews [Jadad or other approach], how they operationalised the Jadad algorithm, steps where they identified discordance, number of included systematic reviews, type of systematic review (e.g. individual patient data meta-analysis, systematic review with narrative summary, systematic review with meta-analysis of RCTs), type of analysis (narrative summary or meta-analysis), risk of bias/quality assessment (e.g. AMSTAR, AMSTAR 2, or ROBIS tool), risk of bias judgments, and whether risk of bias/quality assessment was integrated into the synthesis.
4. Results (results of discordance assessment, effect size and confidence intervals (CIs), number of total participants in treatment and control groups, number of total events in treatment and control groups), direction of study results (favourable or not favourable).
5. Difference between results and conclusions; defined as if these sections disagreed in direction of effect (results, not favourable; conclusions, favourable), authors' result interpretation (quote from abstract and discussion section about the primary outcome result).
6. Categorisation of the condition of the discordant review using 10th revision of the International Statistical Classification of Diseases and Related Health Problems (ICD), a medical classification list by the World Health Organization (WHO) found at https://icd.who.int/browse10/2016/en

Direction of effect and statistical significance
We defined direction of effect and statistical significance according to Jadad's definitions [16], namely:

- Direction of effect: One review favours the intervention, reports null results, or favours the comparison
- Statistical significance indicates the probability associated with the null hypothesis, but it does not determine whether the result is important, meaningful, substantive, large enough to care about, or clinically significant. The p-value incorporates information about both the sample size and effect size, and thus interpretation, comparison of statistical significance across reviews with different sample sizes, and effect estimates is tricky. For example, if one study yielded a p-value of 0.05 and another yielded a p-value of 0.01, then in the absence of any additional information, a knowledge user might assume that the effect size was stronger in the latter case. In fact, though, if the study with a p = 0.05 used a sample of 10 per group and the second (p = 0.01) used 50 per group, then the effect size would have been substantially larger in the study with the modest p-value (a 40-point effect as compared with a 25-point effect).

Systematic review level extraction
The outcomes from section 2.8 were extracted from the discordant review, along with the following information from the included systematic reviews relevant to the primary outcome:

1. Study characteristics (lead author's name, publication year).
2. Research question (objectives, health condition of treatment, PICO eligibility criteria: participant, intervention/comparison, and primary outcome; language restrictions and restrictions on publication status in eligibility criteria; citation of previous systematic reviews/meta-analyses in background or discussion).
3. Search methodology (the name and number of databases searched, grey literature search details, the search period, language restrictions, restrictions on publication status, included full search in an appendix).
4. Methods (number and first author/year of included RCTs, effect metric (OR, RR, MD) and CIs, whether review authors assessed the clinical (PICO) heterogeneity across RCTs (in the methods or results

sections), analysis method (appropriate weighted technique to combine study results [i.e. used a fixed or random random-effects model], investigation of statistical heterogeneity [i.e. by reporting I2, tau2 or chi2], and if heterogeneity is present, then the authors investigated the causes of any heterogeneity [i.e. by reporting subgroup, sensitivity, or meta-regression analyses]), risk of bias/quality assessment (e.g. Cochrane risk of bias tool v1 or v2), risk of bias/quality judgment for each RCT, and whether the RCT quality/risk of bias assessment was integrated into the synthesis; two reviewers independently screened studies, extracted data and assessed risk of bias with process for resolving discrepancies found when comparing.

5. Results (effect size and CI, number of total participants in treatment and control groups, number of total events in treatment and control groups), direction of study results (favourable or not favourable).
6. Difference between results and conclusions; defined as if these sections disagreed in direction of effect (results, not favourable; conclusions, favourable), authors' result interpretation (quote from abstract and discussion section about the primary outcome result).

Appendix C. Deviations to our protocol

Deviations:

- We did not conduct stage 3 screening as the number of studies using Jadad was low (n=21). Stage 3 entailed screened studies based on the authorship team. In the case that the same 'core' authors (first, last, and/or corresponding) conducted two or more of the identified discordant reviews, we included only the most recent of the discordant reviews from the same authors. This step was performed to optimize the breadth of our sample, as author groups tend to use the same methods to assess discordance (e.g. Mascarenhas [18] and Chalmers [15]).
- Interpretation of Jadad algorithm added as an outcome
- Categorisation of the condition of the discordant review using 10th revision of the International Statistical Classification of Diseases and Related Health Problems (ICD)
- Due to the difficulty in coming to consensus on the primary/first outcome cited in a discordant review (Step C), one senior author chose the primary outcome and interventions in the discordant reviews.
- Our pilot testing also revealed that it was difficult to come to consensus on steps C (do the reviews contain the same trials, and step G (do the reviews have the same selection criteria). We therefore decided to do our Jadad assessment in two steps: first independently and in duplicate extract data for steps C and G and then compare results and resolve any discrepancies; and second, extract the remaining steps independently and then compare results and resolve any discrepancies.
- For our efficiency outcome, namely how much time it took to do a Jadad assessment, we only timed our assessment after Steps C and G were completed. We only timed our assessment of Step H and I. We therefore cannot accurately report on time to complete a full Jadad assessment, only a partial assessment.
- We conducted ROBIS assessments on all the systematic reviews included in each of the discordant reviews
- Extraction of whether the included systematic reviews in the discordant reviews were Cochrane or non-Cochrane reviews

Appendix D
Discordant reviews that did not choose one systematic review as the best evidence

Three discordant reviews reported that they used the Jadad algorithm to assess discordance but did not indicate a final decision step and did not choose one or multiple systematic reviews (Vavken [9], Campbell [10], Druyts [11]). These discordant reviews stated several reasons for the inability to choose among the multiple reviews on the same question: (a) too many differences across the systematic reviews making it difficult to compare, and important inconsistencies in the systematic review results leading to uncertainty in the directness of the evidence [10], (b) all studies were of equal quality and built on equal methods [9], and (c) only aimed to assess discordance and not pick one systematic review for a clinical question [11].

Vavken [9] deviated from the Jadad algorithm in several ways. The authors first assessed and ranked the quality of all included systematic reviews using the Oxman-Guyatt tool, and the QUOROM reporting checklist for published systematic reviews. After choosing the highest quality systematic reviews, if these were still conflicting, they evaluated if: (a) the rationale of repeating the meta-analysis, (b) the same studies, (c) same outcomes, and (d) methods for meta-analysis were reported across the systematic reviews. If conflicting results still remained, they assessed the statistical significance and magnitude of the differences in outcomes to test whether conflicts were supported. Vavken [9] found that conflicts derived from interpretation of results rather than from the pooled results, and therefore the discordance could be resolved by assessing whether conclusions were supported by the results.

Two discordant reviews did not report a final decision step to choose one systematic review across multiple discordant systematic reviews (i.e. Bolland [12] and Erickson [13]). Bolland [12] stated that they chose one systematic review based on two criteria: (a) the systematic review only included RCT using unadjusted intention-to-treat analysis, and (b) and the systematic review fulfilled the greatest number of items in the AMSTAR tool. Erikson [13] reported that two out of four included systematic reviews had the highest quality, and were chosen based on the Jadad algorithm, but they did not state which step provided the concluding option.

Appendix E
Case study of the clinical impact of choosing one systematic review using the Jadad algorithm

Discordant review: Houck et al., 2017 [14]
Treatment: Early versus delayed rehabilitation after rotator cuff repair
Primary outcome: Range of motion as assessed with shoulder forward flexion (degrees of forward shoulder rotation)
Minimal clinically important difference (MCID): 12°

Houck et al. [14] analyzed overlapping meta-analyses on the effectiveness of early mobilization compared to delayed rehabilitation after rotator cuff repair. The primary outcome was range of motion where a 12° improvement in forward shoulder flexion was a minimum clinically important difference (MCID). The systematic review (i.e. Riboh 2014 [15]) chosen by discordant review authors [14] after using the Jadad algorithm was different from the one we chose (i.e. Chan 2014 [16]) due to the difference in steps chosen. We judged that the PICO eligibility criteria of the included systematic reviews was similar (Step G), but the discordant review authors [14] disagreed, which led to them moving onto Step I, and not Step H as we had.

The differences in interpretation of the Jadad algorithm between us and the discordant review authors [14] led to divergence in results. The systematic reviews by Riboh 2014 [15] and Chan 2014 [16] show a statistically significant difference for the primary outcome shoulder forward flexion. Riboh 2014 [15] reported a probable clinically important result in forward flexion (MD 14.70, CI 95% 5.52-23.87) considering that effect size exceeds the MCID of 12.0. Chan 2014 [16] reports a result in the same direction (MD 1.05, CI 95% 0.03-2.06) that does not reach clinical significance and does not come close to the MCID.

In the conclusions of both the abstract and the main text, the authors of the discordant review [14] seem to emphasise the adverse effects by reporting that "early motion improves ROM after rotator cuff repair increases the risk of rotator cuff retear"; however, in Riboh [15] and Chan [16] there are no significant differences reported in the risk of rotator cuff retears between delayed and early mobilization.

In post-surgical rehabilitation practice, clinicians have argued over early mobilization to reduce patient deconditioning and longer immobilizations to ensure protection of damaged and repaired tissues. Developed in the last two decades, the Enhanced Recovery After Surgery (ERAS) approach aims for an accelerated and early post-surgical recovery to reduce in-patient stay without increasing complications, reducing the overall costs to the healthcare system. In the wake of this global care pathway, both these systematic reviews report that early rehabilitation is no worse than delayed rehabilitation based on our primary outcome, with the same direction of effect. However, there is still a significant difference in magnitude between effect sizes (MD 14.70 vs MD 1.05) and a clear inconsistency between results with no overlap in confidence intervals. In addition to creating challenges when applying and communicating the effect sizes, the differing estimated effects between these reviews could lead to discordant conclusions on a possible superiority of one approach over the other, originally due to only a different interpretation and application of the Jadad algorithm, which could definitely have an impact on clinical practice.

Appendix F

How easy were the Jadad steps in interpreting and operationalising? (Ease of use)

Step E includes the comparison of clinical heterogeneity, which requires some knowledge on the specific clinical question the study is investigating. The judgement as to what extent the observed clinical differences may result in a heterogeneity is also subjective. Relevant information can be reported in any part of the methods section, results section, and discussion section, dispersively. The information can be hybridised in different paragraphs, so the assessor needs to go through many parts of the text.

Step F is related to the risk of bias of the systematic reviews, which can be reported in the methods section. It is straightforward if any tools are mentioned (e.g., ROBIS, AMSTAR 2), but when no tool was used, multiple tools were used, or the reporting is not detailed or transparent, the authors must assess the quality on their own, which involves more time, resources, and reduces the ease of use.

Step H and I are relatively easy to assess, because they are a yes/no question - a particular sentence/description is reported or not. The items are usually and only reported in the methods section in a clearly structured way. Search strategies (Step H), publication status of the primary trials (Step I) and language restrictions (Step I) can be found in the search methods section. Application of selection criteria (Step H) can be found in the study selection methods section. Quality of the trials (Step I) can be found in the quality assessment section so when assessing these items, we only need to go to these paragraphs or sentences.

Appendix G
Time and ease of use in completing Steps H and I of the Jadad assessments

Of the 18 **Jadad algorithm** assessments completed for Steps H and I, the average time was 60 minutes (Table 1). The **discordant reviews** in Table are listed in the order in which they were assessed. For example, Grassi 2018 [17] was the first **discordant review** assessed after the pilot testing, and Zhao 2015 [18] was the last.

Ten Jadad assessments took between 15 minutes and 47.5 minutes with an average of 4.3 systematic reviews to assess (range 3 - 6). Nine out of the ten were rated as easy to assess, and one was rated as being moderately difficult to assess. These nine easy-rated assessments had evaluated Step H of the Jadad algorithm as the final decision step. The moderately rated assessment had evaluated Step I as the final decision step.

Supplementary Table 1: Time to complete and 'ease of use' ratings for 18 Jadad assessments Step H and I (n = 18)

Overview First Author Year	# of SRs	Jaded final decision step	Reviewer 1 Time in minutes	Reviewer 2 Time in minutes	Average time	Reviewer 1_ Ease of use rating	Reviewer 2_ Ease of use rating	Median Ease of use rating
Chen 2018	5	Step I	45	50	47.5	Moderate	Moderate	Moderate
Chen 2019	4	Step H	65	17	41	Easy	Easy	Easy
Erickson 2015	2	Step H	82	100	91	Moderate	Moderate	Moderate
Fu 2019	4	Step H	50	45	47.5	Easy	Easy	Easy
Grassi 2018	5	Step H	26	40	33	Hard	Hard	Hard
Guo 2018	4	Step H	40	50	45	Easy	Easy	Easy
Houck 2017	5	Step H	76	NR	76	Easy	Easy	Easy
Mascarenhas 2014	3	Step H	46	26	36	Easy	Easy	Easy
Mascarenhas 2015	6	Step H	98	120	109	Moderate	Moderate	Moderate
Pekala 2019	7	Step I	80	65	72.5	Moderate	Moderate	Moderate
Poolman 2007	3	Step H	120	120	120	Moderate	Hard	Mod/Hard
Song 2016	6	Step H	75	63	69	Easy	Easy	Easy
Tan 2018	6	Step H	45	40	42.5	Easy	Easy	Easy
Xing 2016	10	Step H	90	100	95	Moderate	Moderate	Moderate
Xu 2017	5	Step H	60	30	45	Easy	Easy	Easy
Zhao 2015	3	Step H	20	27	23.5	Easy	Easy	Easy
Zhao 2015	4	Step H	15	15	15	Easy	Easy	Easy
Zhiyong 2019	6	Step I	60	78	69	Moderate	Moderate	Moderate

Eight Jadad assessments took between 15 minutes and 120 minutes with an average of 5.6 systematic reviews to assess (range 2 - 10). Two out of the eight were rated as easy to assess, and six were rated as being moderately difficult to assess. The two assessments using Step I as the final decision step were rated as moderate to assess. Four out of the six assessments using Step H as the final decision step were rated as moderately difficult to evaluate, and two were rated as easy to evaluate.

Appendix H
ROBIS Results and Supplementary Table 2

Of 98 systematic reviews, 41 were at low risk of bias, and 57 were at high risk of bias. In 10 discordant reviews, the discordant review authors and ourselves chose the lowest risk systematic review:

- In 7 discordant reviews the choice of systematic review was concordant (Bakdach 2020, Chalmers 2015, Grassi 2018, Erickson 2015, Zhao 2015a, Poolman 2007, Mascarenhas 2015)
- In 3 discordant reviews, the authors and ourselves chose a low risk systematic review, but the systematic review was different (Chen 2019, Zhiyong 2019, Zhao 2015b).

In 4 discordant reviews we chose the lowest risk SR while the authors chose the highest risk SR (Song 2016, Houck 2017, Pekala 2019, Fu 2019). In one of them (Song 2016) the authors chose an additional low-risk SR different from our choice.

In 2 discordant reviews both the authors and we chose the highest risk SR even if there were low-risk SRs included: in one case the SR chosen was the same (Chen 2018), while in the other (Xing 2016) the choice was discordant.

In 3 discordant reviews we chose the highest risk SR while the authors chose the lowest risk SR (Mascarenhas 2014, Tan 2018, Guo 2018).

- In 1 discordant review (Xu 2017) we and the authors chose the same two SRs: one at high risk and one at low risk of bias.
- In 1 discordant review (Bolland 2014) all the SRs included were at high risk of bias and we chose the same SR as the authors.

Overall from the 21 discordant reviews we chose 19 low risk SRs and 9 high risk SRs, while the discordant review authors chose 17 low-risk SRs and 8 high-risk SRs.

Table Legend:
Green = our choice
Pink = Author's choice
Yellow = concurrent choice of review

Supplementary Table 2: ROBIS assessments across discordant reviews

Discordant review Citation	SR First Author, Year	Risk of bias
**Bakdach 2020**	Sardana 2019	Low
	Tasios 2019	Low
	Nascimento 2016	High
**Chalmers 2015**	Pulavarti 2009	Low
	Lenters 2007	Low
**Bolland 2014**	Bischoff-Ferrari 2005	High
	Bischoff-Ferrari 2009	High
	Avenell 2009	High
	Chung 2011	High
**Grassi 2018**	Pakos et al.	High
	Li et al.	Low
	Pavlou et al.	High
	He 2011	Low

Discordant review Citation	SR First Author, Year	Risk of bias
**Xing 2016**	Lo 2003	High
	Wang 2004	High
	Modawal 2005	High
	Arrich 2005	Low
	Strand 2006	High
	Bellamy 2006	High
	Bannuru 2011	High
	Miller 2013	High
	Richette 2015	High
	Strand 2015	High
**Mascarenhas 2015**	Li 2014	Low
	Kongtharvonskul 2013	High
	Li 2013	Low

	Chen et al.	High
**Erickson 2015**	Hing 2011	Low
	Cheng 2014	High
**Chen 2019**	Kanchanatawan 2015	High
	Dai 2016	Low
	Xu 2017	High
	Shen 2017	Low
**Chen 2018**	Li 2013	Low
	Yang 2013	High
	Sun 2016	High
	Huang 2014	High
	Feng 2015	High
**Xu 2017**	Duan 2011	High
	Lenza 2015	Low
	Zhu 2015	High
	Wang 2015	Low
	Hussain 2016	High
**Song 2016**	Siddiqui 2008	High
	Gurusamy 2013	Low
	Zhou 2014	Low
	Cao 2015	High
	Menahem 2015	High
	Wu 2015	Low
**Zhao 2015**	Xu 2013	High
	Lenza 2013	Low
	Kong 2014	High
**Poolman 2007**	Yunes 2001	High
	Forster 2005	High
	Biau 2006	Low
**Mascarenhas 2014**	Millett 2013	Low
	Xu C 2014	High
	Sheibani-Rad 2013	High
**Tan 2018**	Li 2013	High
	Lin 2013	High
	Yang 2013	High
	Feng 2015	Low
	Yang 2017	High
	Huang 2014	High

	Tiamklang 2012	Low
	van Eck 2012	Low
	Meredick 2008	Low
**Houck 2017**	Chen 2015	Low
	Chang 2015	Low
	Chan 2014	Low
	Riboh 2014	High
	Shen 2014	Low
**Pekala 2019**	Chen 2017	High
	Jiang Jiang 2017.	High
	Nong 2016	Low
	Pabalan 2017	High
	Xu 2012	High
	Zhao 2015	High
	Zong 2015	High
**Zhiyong 2019**	Sun 2016	Low
	Feng 2015	Low
	Chen 2014	High
	Huang 2014	Low
	Yang 2013	Low
	Li 2013	Low
**Fu 2019**	Handoll 2012	Low
	Mao 2014	High
	Jia 2014	High
	Rabi 2015	High
**Zhao 2015**	Heineman 2010	Low
	Zheng 2012	Low
	Ouyang 2013	Low
	Bhandari 2006	High
	Ma 2013	Low
**Guo 2018**	Hu 2011	High
	Zhu 2009	High
	Xia 2014	Low
	Xu 2015	High

Appendices references

1. Tricco, A.C., et al., *Non-Cochrane vs. Cochrane reviews were twice as likely to have positive conclusion statements: cross-sectional study.* Journal of Clinical Epidemiology, 2009. **62**(4): p. 380-386.e1.
2. Shea, B.J., et al., *Development of AMSTAR: a measurement tool to assess the methodological quality of systematic reviews.* BMC medical research methodology, 2007. **7**(1): p. 1-7.
3. Shea, B.J., et al., *AMSTAR 2: a critical appraisal tool for systematic reviews that include randomised or non-randomised studies of healthcare interventions, or both.* bmj, 2017. **358**.
4. Whiting P, S.J., Churchill R. *Introduction to ROBIS, a new tool to assess the risk of bias in a systematic review*. in *23rd Cochrane Colloquium*. 2015. Vienna, Austria: John Wiley & Sons.
5. Jadad, A.R., D.J. Cook, and G.P. Browman, *A guide to interpreting discordant systematic reviews.* Cmaj, 1997. **156**(10): p. 1411-6.
6. Viswanathan, M., et al., *Assessing the risk of bias in systematic reviews of health care interventions.* Methods guide for effectiveness and comparative effectiveness reviews [Internet], 2017.
7. Jadad, A.R., D. Moher, and T.P. Klassen, *Guides for reading and interpreting systematic reviews: II. How did the authors find the studies and assess their quality?* Archives of pediatrics & adolescent medicine, 1998. **152**(8): p. 812-817.
8. Schulz, K.F., et al., *Empirical evidence of bias: dimensions of methodological quality associated with estimates of treatment effects in controlled trials.* Jama, 1995. **273**(5): p. 408-412.
9. Vavken, P. and R. Dorotka, *A systematic review of conflicting meta-analyses in orthopaedic surgery.* Clinical Orthopaedics and Related Research®, 2009. **467**(10): p. 2723-2735.
10. Campbell, J., N. Bellamy, and T. Gee, *Differences between systematic reviews/meta-analyses of hyaluronic acid/hyaluronan/hylan in osteoarthritis of the knee.* Osteoarthritis Cartilage, 2007. **15**(12): p. 1424-36.
11. Druyts, E., et al., *Interpreting discordant indirect and multiple treatment comparison meta-analyses: an evaluation of direct acting antivirals for chronic hepatitis C infection.* Clin Epidemiol, 2013. **5**: p. 173-83.
12. Bolland, M.J. and A. Grey, *A case study of discordant overlapping meta-analyses: vitamin d supplements and fracture.* PLoS One, 2014. **9**(12): p. e115934.
13. Erickson, B.J., et al., *Does operative treatment of first-time patellar dislocations lead to increased patellofemoral stability? A systematic review of overlapping meta-analyses.* Arthroscopy: The Journal of Arthroscopic & Related Surgery, 2015. **31**(6): p. 1207-1215.
14. Houck, D.A., et al., *Early versus delayed motion after rotator cuff repair: a systematic review of overlapping meta-analyses.* The American journal of sports medicine, 2017. **45**(12): p. 2911-2915.
15. Riboh, J.C. and G.E. Garrigues, *Early passive motion versus immobilization after arthroscopic rotator cuff repair.* Arthroscopy: the journal of arthroscopic & related surgery, 2014. **30**(8): p. 997-1005.
16. Chan, K., et al., *Delayed versus early motion after arthroscopic rotator cuff repair: a meta-analysis.* Journal of shoulder and elbow surgery, 2014. **23**(11): p. 1631-1639.
17. Grassi, A., et al., *Patellar resurfacing versus patellar retention in primary total knee arthroplasty: a systematic review of overlapping meta-analyses.* Knee Surgery, Sports Traumatology, Arthroscopy, 2018. **26**(11): p. 3206-3218.
18. Zhao, J.G., et al., *Intramedullary nail versus plate fixation for humeral shaft fractures: a systematic review of overlapping meta-analyses.* Medicine (Baltimore), 2015. **94**(11): p. e599.
